# Afforestation as a mitigation strategy: countering climate-induced risk of forest carbon sink in China

**DOI:** 10.1186/s13021-025-00308-1

**Published:** 2025-06-21

**Authors:** Yuan Cao, Deyu Zhong, Rong Shang, Qihua Ke, Mingxi Zhang, Di Xie, Shutong Liu, Chensong Zhao, Randongfang Wei

**Affiliations:** 1 State Key Laboratory of Hydroscience and Engineering, Tsinghua University, Beijing, 100084 China; 2 Key Laboratory of Hydrosphere Sciences of the Ministry of Water Resources, Tsinghua University, Beijing, 100084 China; 3 Department of Hydraulic Engineering, Tsinghua University, Beijing, 100084 China; 4 Key Laboratory of Humid Subtropical Eco-Geographical Process of Ministry of Education, School of Geographical Sciences, Fujian Normal University, Fuzhou, 350117 China

**Keywords:** Forest carbon sink, China, Afforestation, Growth model, Climate change

## Abstract

**Background:**

China has made substantial efforts in afforestation since the 1970s, significantly contributing to the country’s forest carbon sink. However, the future carbon sink dynamics remain uncertain due to anticipated changes in forest age structure, climate conditions, and atmospheric CO_2_ concentrations. Moreover, the extent to which afforestation can enhance future carbon sequestration has not been fully quantified. This study focuses specifically on China and integrates forest growth models with Maximum Entropy (MaxEnt) models to project future carbon dynamics based on shifts in forest habitat suitability. A nature scenario is applied to evaluate potential climate-induced risks to forest carbon sequestration, while an afforestation scenario is used to assess the additional contribution from planned afforestation efforts.

**Results:**

The baseline aboveground biomass (AGB) of China’s forests in 2020 is estimated at 11.59 ± 4.06 PgC. Under the nature scenario and assuming no future disturbances, the total AGB is projected to increase by 5.20–5.74 PgC by the 2050s and by 6.35–8.11 PgC by the 2070s, while carbon sequestration rates are expected to decline from 146.03 to 165.03 TgC/yr to approximately 122.98–137.80 TgC/yr. Between 11.79 and 39.60% of forests are at risk of land loss and compositional shifts in the 2070s, with the situation exacerbated under the SSP585 scenario. To mitigate climate-induced risks, the afforestation scenario proposes an additional 117.90–129.32 Mha of suitable forest area by the 2070s. Newly planted forests are projected to contribute approximately 37.42–65.60% of the carbon sequestration achieved by existing forests during the same period.

**Conclusions:**

Climate change is projected to cause significant forest loss and compositional changes across China. Although total forest carbon storage is expected to increase, the overall rate of carbon sequestration will likely decline. Afforestation emerges as a key strategy to enhance future forest carbon sinks. This study provides a spatially explicit assessment of carbon sequestration potential through afforestation and offers science-based guidance for the design of targeted forest policies in China.

**Supplementary Information:**

The online version contains supplementary material available at 10.1186/s13021-025-00308-1.

## Background

Terrestrial ecosystems are major carbon sinks, absorbing nearly one-third of total anthropogenic CO_2_ emissions [16]. Forests, in particular, play a central role in the carbon cycle and contribute approximately 80% of the carbon sinks among terrestrial ecosystems [41]. Consequently, enhancing forest carbon sink is recognized as a crucial strategy for mitigating climate change risks [31].

At the same time, climate change has exerted multifaceted effects on forest ecosystems. Empirical studies suggest that global warming, intensified water stress, and the increasing frequency of disturbances such as wildfires and insect outbreaks can contribute to forest degradation and reductions in carbon storage [34], whereas elevated atmospheric CO_2_ concentrations have been associated with enhanced forest productivity [2]. Beyond climatic influences, intensive anthropogenic land-use changes, including urban expansion [59], agricultural conversion [50], and commercial harvesting [23], have significantly reduced forest cover and altered carbon accumulation patterns. According to the Food and Agriculture Organization [14], the global forest area declined from 32.5 to 30.8% of the total land area between 1990 and 2020. During this period, approximately 420 Mha of forest were converted to other land uses, and the area of primary forest decreased by over 8 Mha worldwide.

Afforestation and reforestation are widely recognized strategies for counteracting the global decline in forest cover. Reforestation refers to restoring forests on land that was forested within the past 50 years, while afforestation refers to establishing forests on land that has not supported forest cover for more than 50 years [20]. This study encompasses both practices but does not distinguish between them; for simplicity, the term"afforestation"is used throughout. Numerous large-scale forest afforestation initiatives have been launched globally to expand forest area [6]. Notably, over half of the countries that signed the Paris Agreement included afforestation as a key component of their Nationally Determined Contributions to climate change mitigation [46].

China plays a pivotal role in global carbon dynamics, owing to its historically high levels of anthropogenic disturbance and its current status as one of the largest emitters of carbon dioxide, driven by rapid industrialization. In response to growing concerns over climate change, China has pledged to achieve carbon neutrality by 2060 [30]. As part of its mitigation strategy, the Chinese government has implemented a series of large-scale afforestation and reforestation programs since the 1970 s, aimed at converting croplands and marginal lands into forests. These efforts have resulted in a high afforestation rate, with approximately 1.7 Mha of planted forest annually over the past two decades [38]. By 2008, China had the largest area of planted forests in the world, covering about 62 Mha, which represented 23% of the global plantation area [15]. The expansion of forest cover has significantly enhanced terrestrial carbon sequestration capacity. For example, the Natural Forest Conversion Program alone contributed an estimated 0.45 PgC in biomass accumulation between 1980 and 1998, offsetting approximately 28–37% of fossil fuel emissions during that period [13, 42]. More broadly, forest expansion has been attributed to nearly 44% of China’s total terrestrial carbon sink from 1980 to 2019 [65].

Due to afforestation’s notable role in enhancing carbon stocks, it has been recognized as a viable nature-based solution for climate change [47]. To enhance the forest carbon sink, China has established several afforestation targets for the future. For instance, the Action Plan for Carbon Dioxide Peaking Before 2030 [52] sets a goal to increase forest cover to 25% by 2030. Therefore, projections of future forest carbon sinks in China must explicitly account for national afforestation targets. This study aims to address two key questions: (1) How will climate change affect China’s forest carbon sink in the absence of human intervention? and (2) To what extent can afforestation contribute to enhancing carbon sequestration under China’s afforestation commitments? To answer these questions, this study integrates forest growth models with Species Distribution Models (SDMs) to first simulate a baseline scenario without human disturbances, thereby assessing climate-driven risks to forest carbon sinks, such as potential losses due to reductions in forest area and shifts in species composition. We then evaluated an afforestation scenario to quantify the additional carbon sequestration benefits resulting from increased forest cover.

## Methods

### Data descriptions

#### Forest field survey data

In this study, biomass-age statistical growth models are fitted using the field measurement dataset developed by Luo et al. [32]. This dataset compiles published data in China from 1978 to 2008, encompassing 348 study sites with information on site location, forest type, biomass, stand age, as well as mean annual temperature (MAT) and mean annual precipitation (MAP) as climatic data. Adhering to data collection and quality control criteria from Luo et al. [32], we supplement the dataset with additional data from recent literature and extend the time period to 2020, resulting in a total of 1,695 measurements. This dataset is openly available at https://doi.org/10.6084/m9.figshare.27130098.v1.

To align with the forest distribution map's forest types, the data are categorized into evergreen needle-leaved forest (ENF), deciduous needle-leaved forest (DNF), evergreen broad-leaved forest (EBF), deciduous broad-leaved forest (DBF), and mixed forest (MF). Given the significant impact of environmental and climatic factors on forest growth, these five forest types are further classified according to seven climatic zones in China. Figure S1 illustrates the distribution of our data points and the division of climatic zones in China. Therefore, 32 sub-forest types are derived. For simplicity and to ensure adequate sample sizes for fitting models, similar forest types are aggregated, resulting in 15 primary forest types, as detailed in Table S1.

#### Climate data

In the forest field survey dataset, MAT and MAP data are sourced from original studies that have passed quality control procedures. For sites lacking reliable climatic data, MAT and MAP values are extracted from a global climate map with a 30 arc-second (~ 1 km) resolution provided by WorldClim (http://www.worldclim.org). WorldClim’s data are generated through the interpolation of average monthly climatic records spanning from 1950 to 2000.

To forecast future biomass, MAT and MAP data are again obtained from 30 arc-second resolution global climate maps from WorldClim (http://www.worldclim.org). We use ensemble projections of bioclimatic variables, averaged across 13 General Circulation Models (GCMs) from the Coupled Model Intercomparison Project Phase 6 (CMIP6). These projections are considered under various climate change scenarios (SSP245 and SSP585) for the time periods corresponding to the 2030 s, 2050 s, and 2070 s (abbreviation for periods of 2021–2040, 2041–2060, and 2061–2080, respectively). Detailed information is listed in Table S2.

#### Forest cover type map

This study utilizes three forest cover type products: GLC_FCS30 [70], CLASS-GLC [29], and ESA CCI LC data [12]. The GLC_FCS30 product, created using time series of Landsat imagery and high-quality training data from the Global Spatial Temporal Spectra Library (GSPECLib) on the Google Earth Engine platform, represents the first global land-cover dataset with a fine classification system and high accuracy at a 30 m resolution. CLASS-GLC, which integrates sample migration, machine learning, and spatio-temporal adjustment, produces a 30 m resolution global land cover map with a 10% accuracy improvement over existing datasets such as Globeland30, based on validation samples from FLUXNET sites. The ESA CCI-LC project provides annual global land cover maps at a 300 m resolution, known for its detailed classification and high accuracy.

In this study,"forest"is defined as grid cells with a tree cover percentage of 20% or greater, consistent with the national survey [39]. To ensure consistency, all datasets are resampled to a 30 arc-second (~ 1 km) resolution. A robust forest cover type composite is constructed using the framework proposed by Shang et al. [48]. The merging process follows these criteria: (1) if at least two datasets agree on the forest cover type for a given pixel, that classification is adopted, (2) in case of discrepancies, GLC_FCS30 is prioritized due to its superior general performance; (3) if GLC_FCS30 classifies a pixel as non-forest, CLASS-GLC is consulted due to its superior resolution compared to ESA CCI; (4) if both GLC_FCS30 and CLASS-GLC classify a pixel as non-forest, the ESA CCI LC classification is used. The resulting forest cover type maps at a 30 arc-second resolution are depicted in Figure S2.

According to the “Report on China's Forest Resources (2014–2018)” published by the National Forestry and Grassland Administration in China, the total arbor forest area is reported to be 179.89 Mha, while our map estimates it at 164.55 Mha. Although our map underrates the area of arbor forest to some extent, the proportions of broad-leaved and needle-leaved forest are similar to the statistical data (as shown in Table S3).

#### Forest age map

In this study, we integrate three distinct sources of forest age data, including a 2020 forest age map developed by Cheng et al. [11], an annual forest age dataset from 1986 to 2022 created by Shang et al. [49], and the MPI-BGC global forest age map from Besnard et al. [4].

Cheng et al. [11] introduced a novel framework combining machine learning algorithms with remote sensing time series analysis to estimate forest age across China. Their approach, validated against independent field plots, achieved an R^2^ value ranging from 0.51 to 0.63, indicating a moderate to strong correlation with empirical data. Shang et al. [49] developed a dataset by integrating forest disturbance detection using Landsat data with age mapping of undisturbed forests through machine learning techniques. This dataset provides annual forest age estimates at a 30-m resolution from 1986 to 2022. For this study, we specifically utilized the forest age map for the year 2020 from Shang et al. [49]. The MPI-BGC map offers a global distribution of forest age around 2010, estimated using a machine learning approach trained on over 40,000 plots with forest inventory, biomass, and climate data.

All maps are resampled to a 30 arc-second (~ 1 km) resolution for consistency. For each grid cell designated as"forest"in the forest cover map, the corresponding forest age is first extracted from Cheng’s forest age map due to its high resolution and robust alignment with field surveys. Null values in Cheng's map are subsequently filled using Shang’s forest age map, which, while specific to China, only includes data for undisturbed forests. Finally, any remaining null values are addressed using the MPI-BGC global forest age map from 2010, with the forest age values adjusted by adding 10 years.

### Statistical models for estimating forest carbon sink

To project forest carbon dynamics in China, we assume that AGB is influenced additively by forest aging, climate change, and atmospheric CO_2_ concentration:

1 $$C = C^{age} + C^{climate} + C^{{co_{2} }}$$

To quantify the impact of forest age on AGB, we apply three different age-biomass growth models, including Michaelis–Menten (MM), Logistic (L), and Monomolecular (MO) model. Following are the introduction of three growth models:

1. Michaelis–Menten growth model [35]:
  2 $$AGB=\frac{\mu t}{k+t}$$
  where $\mu$ is the asymptote for saturated AGB; $\text{k}$ is the age when the system reaches the half of maximum ($\frac{\mu }{2}$).
2. Monomolecular Function [67]:
  3 $$AGB = \mu \left( {1 - ce^{ - \alpha t} } \right)$$
  where $\upmu$ is the asymptote for saturated AGB, α and c determines the rate to reach the asymptote (in this study, $\text{c}\ge 1$).
3. Logistic Function [33]:
  4 $$AGB = \frac{\mu }{{(1 + ce^{ - \alpha t} )}}$$
  where $\upmu$ is the asymptote for saturated AGB, α and c determines the rate to reach the asymptote (in this study, $\text{c}\ge 1$).

Previous studies [17, 64, 74] identified MAT and MAP as significant drivers affecting net ecosystem productivity (NEP). Accordingly, we extend this method to estimate AGB. We add a linear function after the growth function to take climate factors into consideration:

5 $${C}_{t}^{age+climate}=f(t) + a\times MAT+b\times MAP+d$$

where f(t) is the growth function mentioned above; d is the intercept, and a and b are the coefficients for MAT and MAP, respectively.

For simplicity, forest field survey datasets are categorized into 15 primary forest types and fitted using three distinct growth models. For each forest group, we apply these three growth models and assess their performance using three key indicators: the root mean square error (RMSE), the coefficient of determination (R^2^) and the Akaike information criterion (AIC). RMSE and R^2^ serve as metrics for evaluating model errors [9], and AIC measures the accuracy and complexity of the model comprehensively [51]. A thorough comparison of these three indicators is conducted for each forest type, allowing us to identify the most suitable model to describe the age-biomass relationship. The results are summarized in Table 1, and detailed information for all fitted models is presented in Figure S3.

**Table 1:** The sample sizes, the parameter values fitted by growth models and the goodness of fit values (R^2^, AIC) of 15 main forest types defined in this study

Forest type	Size	Model	RM–SE	R^2^	AIC	Growth model function	Parameters for climate factors		
							a	b	d
DBF in north subtropical humid region	66	L	36.9	0.58	488.3	$\frac{86.59}{1+{8.4\times {10}^{5}7\text{e}}^{-0.88\text{t}}}$	−6.14	0.04	91.63
DBF in plateau temperate semi-arid region	24	MM	26.3	0.89	168.9	$59.98\frac{\text{t}}{357.71+\text{t}}$	0.093	− 170.86	206.63
DBF in tropical humid region	21	MO	12.2	0.74	115.2	$205.882\left(1-{\text{e}}^{-0.30\text{t}}\right)$	−31.61	− 0.05	574.91
DBF in warm/mid-temperate region	132	MM	30.1	0.52	909.1	$79.98\frac{\text{t}}{228.93+\text{t}}$	−1.89	70.45	106.18
DNF	159	MM	22.2	0.40	995.3	$58.70\frac{\text{t}}{167.44+\text{t}}$	−0.98	0.04	− 27.41
EBF in plateau semi-arid region	19	MM	18.0	0.67	118.0	$7.27\frac{\text{t}}{193.04+\text{t}}$	−4.51	− 0.02	36.09
EBF in tropical humid region	154	MM	26.7	0.57	1021.8	$21.43\frac{\text{t}}{218.53+\text{t}}$	0.54	− 0.01	10.34
EBF in temperate/subtropical humid/sub-humid region	112	MM	59.1	0.34	923.36	$84.95\frac{\text{t}}{343.12+\text{t}}$	2.01	0.04	− 54.96
ENF in mid-temperate arid/semi-arid region	68	MM	19.9	0.81	416.7	$47.51\frac{\text{t}}{406.95+\text{t}}$	6.36	0.20	− 223.52
ENF in mid-temperate sub-humid region	68	MM	32.9	0.71	485.3	$3.33\frac{\text{t}}{1000.00+\text{t}}$	2.80	0.19	− 941.05
ENF in north subtropical humid region	595	MM	37.9	0.59	4335.4	$49.91\frac{\text{t}}{442.58+\text{t}}$	3.38	0.02	− 113.53
ENF in plateau temperate semi-arid region	36	MM	29.2	0.84	252.9	$44.43\frac{\text{t}}{288.43+\text{t}}$	3.70	0.06	− 91.78
ENF in tropical humid region	59	MM	19.1	0.71	357.8	$40.63\frac{\text{t}}{273.84+\text{t}}$	6.12	− 0.02	− 106.07
ENF in warm temperate sub-humid region	109	L	15.3	0.42	607.1	$\frac{49.54}{1+{2.1\times {10}^{5}\text{e}}^{-0.72\text{t}}}$	0.72	− 0.81	− 0.01
MF	72	MM	13.0	0.74	348.9	$9.48\frac{\text{t}}{176.50+\text{t}}$	1.99	0.01	− 90.84

To quantify the effect of changing forest age and climate separately on AGB, we suppose ${C}_{t}^{age}$ can be calculated by forest age in the future and climate factors in 2020:

6 $${C}_{t}^{age}=f(t) + a\times {MAT}_{2020}+b\times {MAP}_{2020}+d$$

Therefore, ${C}_{t}^{climate}$ can be extracted as the following equation:

7 $${C}_{t}^{climate}={C}_{t}^{age+climate}-{C}_{t}^{age}$$

To estimate the effect of CO_2_ concentration on AGB, we utilize the results from the 1 pctCO2 biogeochemical diagnostic experiment of 12 ESMs from CMIP6 [2], and the detailed information is listed in Table S2. The prediction of ${\text{C}}^{{\text{co}}_{2}}$ follows the method of Xu et al. [58]. For each grid cell, we first determine the CO_2_ concentration evolution under different climate change scenarios (SSP245, SSP585) and extract the corresponding vegetation biomass carbon density (cVeg) from the 1 pctCO_2_ experiment results. We calculate the ratio (F) of simulated forest biomass carbon densities under varying CO_2_ concentrations for future years (i.e., 2020, 2021,…, 2080) relative to the baseline CO_2_ concentration from 1978 to 2020 (371.8 ppm), as follows:

8 $$F=\frac{{C}_{t}^{{co}_{2}}}{{C}_{t0}^{{co}_{2}}}$$

where ${\text{C}}_{\text{t}}^{{\text{co}}_{2}}$ denotes the simulated cVeg corresponding to the mean CO_2_ concentration for year t, and ${\text{C}}_{\text{t}0}^{{\text{co}}_{2}}$ denotes that in the baseline year. The ratios $\text{F}$ for the 2030 s, 2050 s, and 2070 s are averaged from the calculations for the periods 2021–2040, 2041–2060, and 2061–2080, respectively.

Finally, we compute the effect of increasing CO_2_ concentration on AGB using the derived ratios $\text{F}$:

9 $${C}^{{co}_{2}}=(F-1)\times {C}^{age}$$

### Predict forest suitable habitat

In this study, we use MaxEnt model (version 3.4.1), one of the most popular SDMs due to its superior predictive accuracy and user-friendly software [36], to access the current and future potential geographical distributions of 32 sub-forest types by establishing correlations between specie existence patterns and environmental characteristics [1].

Due to the lack of sufficient field survey data, we input the forest cover type map as occurrence points. To enhance the spatial independence of these points, we first rarefy the occurrence data using SDMToolbox [5]. Subsequently, we select 35 environmental variables as input data for modeling the potentially suitable habitats of 32 sub-forest types, including 19 bioclimatic variables, 2 topographical variables, and 14 soil variables. Specifically, the bioclimatic variables are sourced from WorldClim (https://worldclim.org/data/index.html). The elevation data are obtained from the ETOPO Global Relief Model (version: ETOPO 2022) (https://www.ncei.noaa.gov/products/etopo-global-relief-model), and the slope variable is derived from the elevation data using ArcGIS 10.8.1. Soil variables are acquired from the Harmonized World Soil Database (HWSD v 1.21) (http://www.iiasa.ac.at/web/home/research/researchPrograms/water/HWSD.html). All datasets are at a spatial resolution of 30 arcseconds (~ 1 km). To mitigate the influence of collinear variables on the accuracy of SDMs [5], a correlation analysis is conducted to exclude variables with high correlation coefficients ($|\text{r}|>0.8$). As a result, 8 bioclimatic variables, 2 topographical variables, and 7 soil variables are selected for this study. Detailed information on these variables is provided in Table S4.

We carry out MaxEnt models 10 times for each sub-forest type to enhance robustness, and the results are finally averaged. The maximum number of background points is set to 10,000, and the maximum number of iterations is capped at 5,000, while other parameters are kept at their default settings. Model accuracy is evaluated using the Area Under the Curve (AUC) of the Receiver Operating Characteristic (ROC) curve, with values typically ranging from 0.5 to 1.0. The results show that the average AUC for the models of the 32 sub-forest types is 0.89 ± 0.11 (shown in Table S1), indicating that our SDMs possess strong predictive capability, as a model is considered well-performing when it achieves an AUC greater than 0.78 [54].

To predict the potential suitable habitats in the future, we select the ensemble projections of bioclimatic variables averaged across 13 GCMs of the CMIP6 under different climate change scenarios (SSP245, SSP585) for the periods 2030 s, 2050 s and 2070 s, which are downloaded from WorldClim, and the changes of 8 bioclimatic variables in future are shown in Figure S4-S11. Additionally, we assume that the two topographical variables and seven soil variables remain unchanged over the next 60 years. We use all data to project maps of potential suitable habitats in the future.

### Project future carbon sink dynamics

Considering China has set policy goals for future forest areas, we assume two different scenarios for prediction: the nature scenario and the afforestation scenario.

#### Nature scenario

In this scenario, we assume no further disturbances, such as natural disasters, human deforestation and afforestation. The existing forest will naturally die or be replaced by other tree species due to changes of forest suitable habitat. We estimate the future carbon sink following these steps: firstly, future suitability probability (P) for each pixel and the suitability threshold of the maximum training sensitivity plus specificity (MTSS) [28] for each sub forest type are extracted from MaxEnt model outputs (MTSS values are shown in Table S1). Secondly, for a pixel supporting forest type A with stand age t in 2020, if P_future_ exceeds MTSS after N years, this pixel is assumed to remain suitable for forest type A. The growth model is then extrapolated with the extended stand age (t + N) and future climate data to estimate AGB in the future. If P_future_ is lower than MTSS, we extract P_future_ for other forest types on this pixel and assume that forest type B, with the highest P_future_ and meeting the forest type existence condition (P > MTSS), will replace the current type, with a stand age of N/2.

#### Afforestation scenario

As shown in Figure S12 and Table S5, future national forest area targets increase linearly over time. Across linear regression, we interpolate the forest area target for each time period (2030s, 2050 s, 2070 s). For each period, we first access the results under the nature scenario and calculate the forest area. Next, we derive the afforestation area by subtracting the existing forest area under the nature scenario from the forest area target. Thirdly, we randomly select pixels nationwide; for each pixel, we select the forest type with maximum P_future_ that satisfies forest type existence condition (P > MTSS), and assume the stand age of newly planted forest is N/2. We repeat this procedure 10 times and average the results to enhance robustness.

## Results

### Estimation of forest carbon sink in 2020

The growth models for 15 primary forest types are detailed in Table 1. The average AGB estimated by our models for China is 114.63 ± 40.12 Mg/ha (mean ± standard deviation, hereafter). The total AGB is estimated to be 11.59 ± 4.06 PgC, accounting for age, climate, and CO_2_ parts.

**Fig. 1:**
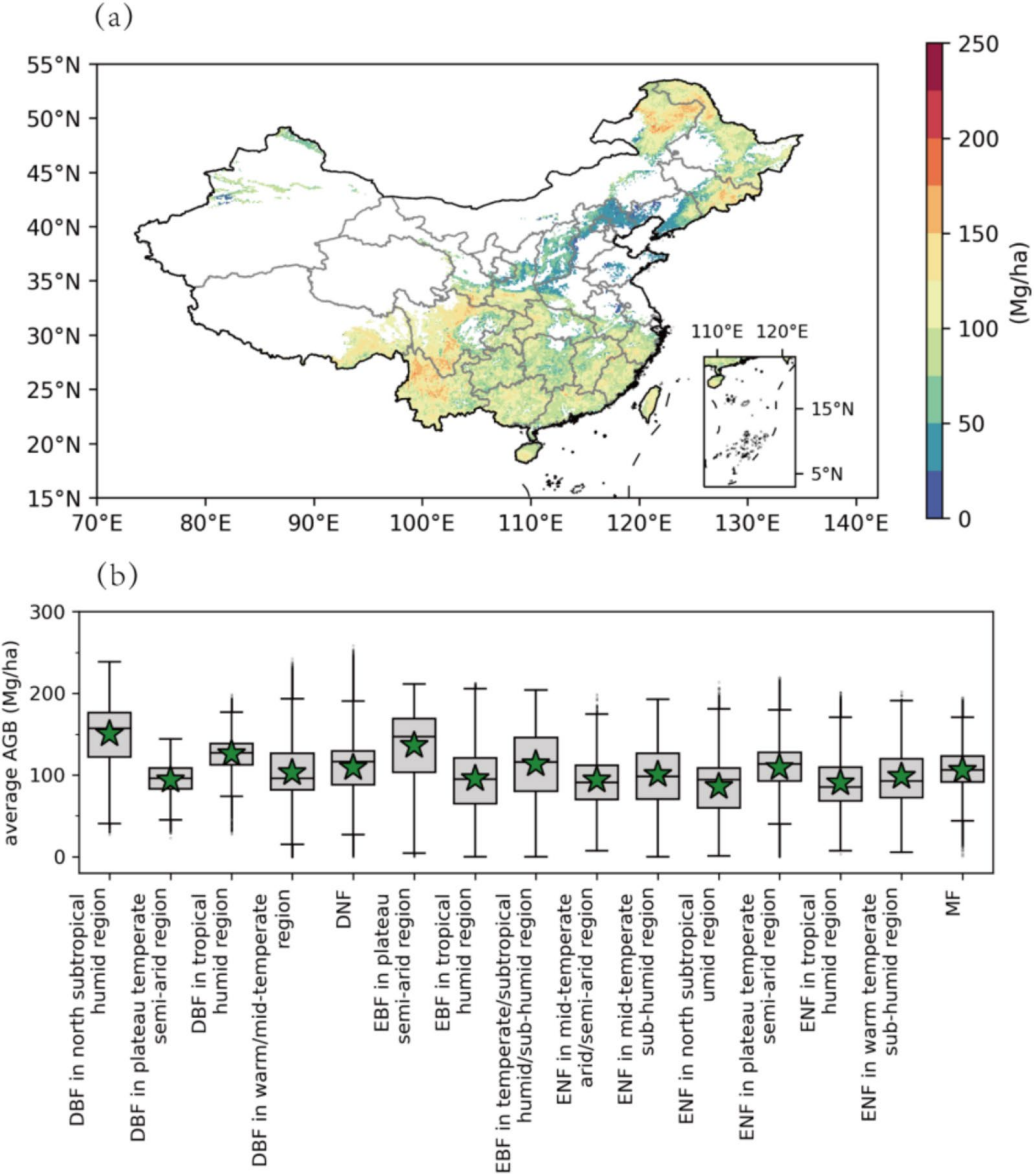
**a** Estimated spatial distribution of AGB (Mg/ha) in 2020 (averaged at a spatial resolution of 0.1°); **b** average AGB (Mg/ha) across 15 forest types (green stars represent average values)

Figure 1 shows the spatial distribution of estimated AGB in China and the AGB estimates across 15 main forest types. Forests in the central China exhibit the highest biomass density (> 100 Mg/ha), whereas the central-western region and northern-eastern region show the relatively low biomass density. Most of the western region is considered unsuitable for forest growth.

Variations in AGB accumulation are evident across the 15 forest types. DBF in north subtropical humid region and EBF in plateau/mid-temperate semi-arid region exhibit the highest AGB, estimated at 150.45 ± 35.78 Mg/ha and 135.95 ± 39.83 Mg/ha, respectively. In contrast, ENF groups exhibit relatively low biomass compared to broad-leaved groups, for example, the average AGB of ENF in north subtropical humid region is estimated at 85.99 ± 29.91 Mg/ha. Among all forest types, DBF in warm/mid-temperate region dominates, comprising 25.14% of the total forest area, with a biomass density estimated at 102.98 ± 30.77 Mg/ha.

### Projected forest carbon sink under climate change

**Fig. 2:**
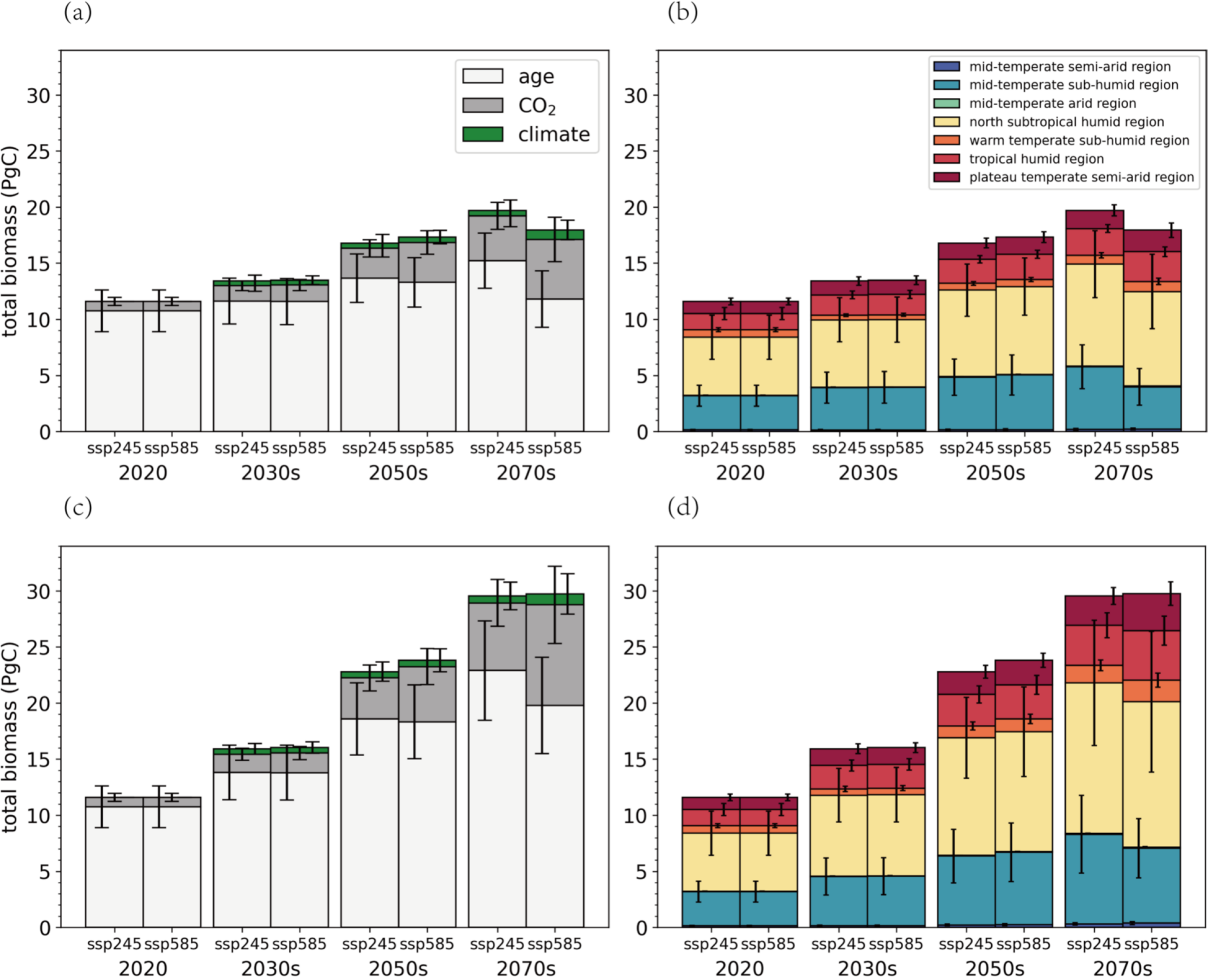
Prediction of total AGB (PgC) under (**a**, **b**) the nature scenario and (**c**, **d**) the afforestation scenario. In (**a**, **c**), the total AGB estimations are partitioned into age, CO_2_ part and climate part; while in (**b**, **d**) are segmented by different climate regions. Black lines represent standard deviations

In the nature scenario, there is no afforestation under consideration, and the distribution of forest is decided by changes in suitable habitats. Figure 2 and Table 2 illustrate projected total AGB under the SSP245 scenario, reaching 13.42 ± 4.60 PgC in the 2030 s, 16.79 ± 5.40 PgC in the 2050 s, and 19.70 ± 6.65 PgC in the 2070s. These values correspond to increases of approximately 1.83, 5.20, and 8.11 PgC relative to the 2020 level. Under the SSP585 scenario, the total AGB is projected to reach 13.49 ± 4.69 PgC, 17.33 ± 5.87 PgC, and 17.94 ± 7.68 PgC in the 2030 s, 2050 s, and 2070 s, representing respective increases of 1.90, 5.74, and 6.35 PgC from 2020. Under the SSP245 scenario, the forest age effect predominates, contributing 86.66%, 81.37%, and 77.29% of the total AGB in the 2030 s, 2050 s, and 2070 s, respectively. However, the corresponding proportions of ${\text{C}}^{\text{age}}$ are relatively low under the SSP585 scenario due to the increasing CO_2_ fertilization effect, and ${\text{C}}^{{\text{co}}_{2}}$ contributes 29.63% of the total AGB in the 2070 s, which is 9.41% higher than that under the SSP245 scenario. ${\text{C}}^{\text{climate}}$ contributes the least, accounting for only 2% to 5%, and is of great uncertainty.

**Table 2:** Variations of total AGB, stand age, forest area loss rate, forest structural change rate and average AGB in the nature scenario

Period	Climate scenario	Total AGB (PgC)	Stand age (yrs)	Forest area loss rate (%)	Forest structural change rate (%)	Average AGB (Mg/ha)
2020	/	11.59 ± 4.06	57.57 ± 32.21	/	/	114.63 ± 40.12
2030s	SSP245	13.42 ± 4.60	53.40 ± 27.53	0.41	4.42	133.27 ± 45.85
	SSP585	13.49 ± 4.69	53.27 ± 27.68	0.55	4.89	134.16 ± 46.66
2050s	SSP245	16.79 ± 5.40	69.01 ± 28.20	1.27	6.78	168.20 ± 54.10
	SSP585	17.33 ± 5.87	68.28 ± 28.88	2.55	9.32	175.88 ± 59.58
2070s	SSP245	19.70 ± 6.65	84.50 ± 28.85	2.51	9.28	199.87 ± 67.51
	SSP585	17.94 ± 7.68	71.43 ± 29.93	9.45	30.12	196.10 ± 83.95

A decreasing trend in the carbon sequestration rate is observed. For instance, under the SSP245 scenario, the carbon sequestration rate reaches 153.51 ± 267.98 TgC/yr in the 2030 s, decreasing to 137.80 ± 67.41 TgC/yr by the 2070s. Variation across climate change scenarios is noted. Due to the increasing CO_2_ fertilization effect, the carbon sequestration rate under SSP585 scenario is relatively higher in the future, estimated at 165.03 ± 103.70 TgC/yr in the 2050 s compared to 146.03 ± 98.68 TgC/yr for SSP245.

**Fig. 3:**
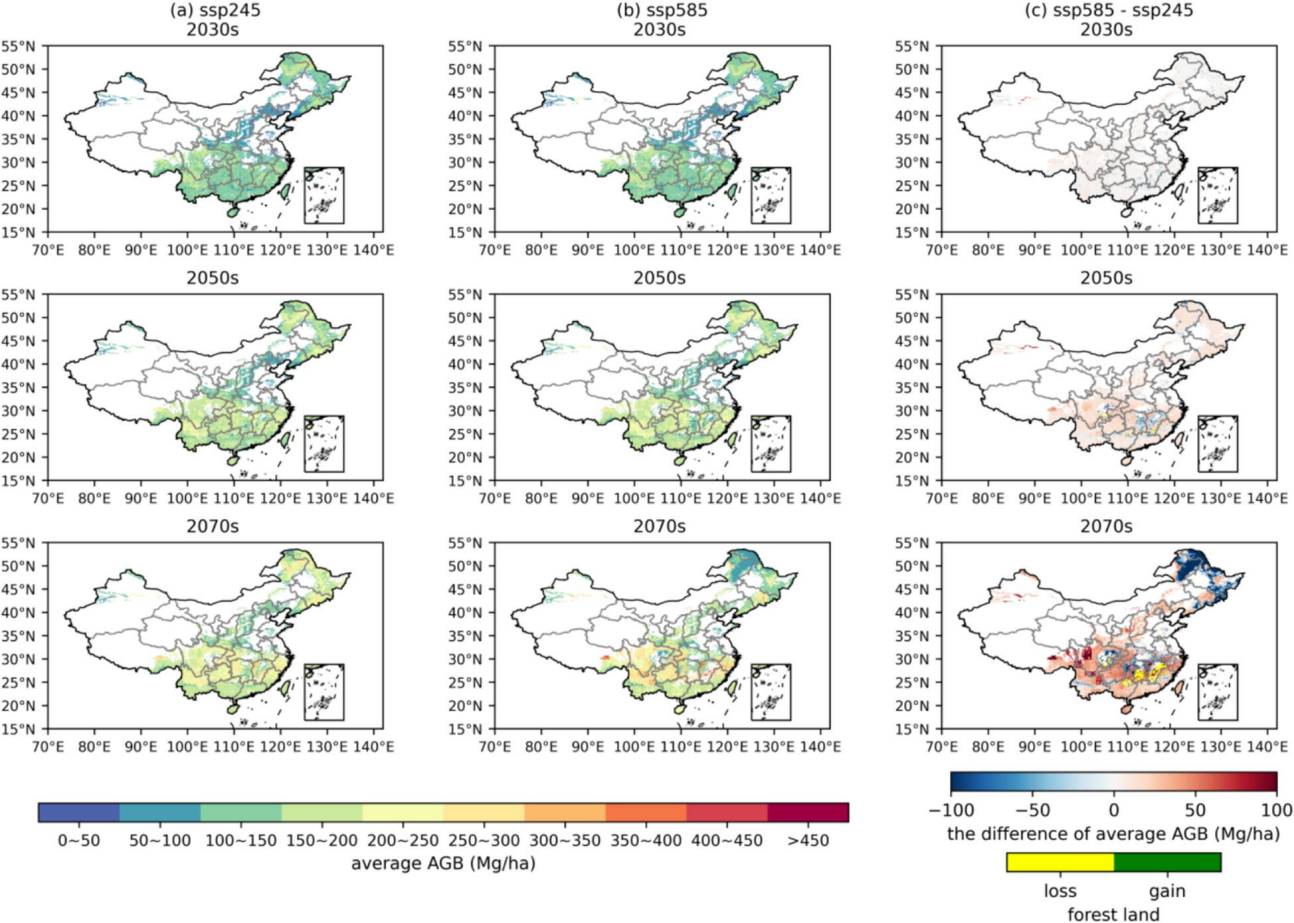
Predictions of AGB’s (Mg/ha) spatial distributions under **a** the SSP245 scenario and **b** the SSP585 scenario; **c** differences between SSP585 and SSP245 scenario (SSP585-SSP245). The blue-red colorbar indicates differences of average AGB (Mg/ha) and the yellow-green colorbar indicates difference of forest land. All maps are averaged at a spatial resolution of 0.1° and under the nature scenario

Figure 3 illustrates the spatial distribution of average AGB under the natural scenario. The average AGB ranges from 133.27 ± 45.85 Mg/ha to 199.87 ± 67.51 Mg/ha across the 2030 s to 2070 s under the SSP245 scenario, and that of SSP585 ranges from 134.16 ± 46.66 Mg/ha to 196.10 ± 83.95 Mg/ha. Overall, forests in the north subtropical humid region are the primary contributors to carbon sequestration, accounting for nearly 45% of the total, whereas forests in the warm temperate sub-humid region contribute the least, with approximately 0.3%. Substantial variations in average AGB are observed in the north subtropical humid and mid-temperate sub-humid regions under different climate change scenarios, as illustrated in Fig. 3. These fluctuations are closely linked to the sharply increasing forest area loss rate and forest structural change rate under the SSP585 scenario in the 2070s. During this period, forests exhibit pronounced vulnerability, with 30.12% undergoing structural changes and 9.45% experiencing direct mortality, as shown in Figure S13 and Table 2. In contrast, the corresponding rates under the SSP245 scenario for the same period are significantly lower, at 9.28% and 2.51%, respectively. These drastic forest dynamics directly contribute to the differences in AGB.

**Fig. 4:**
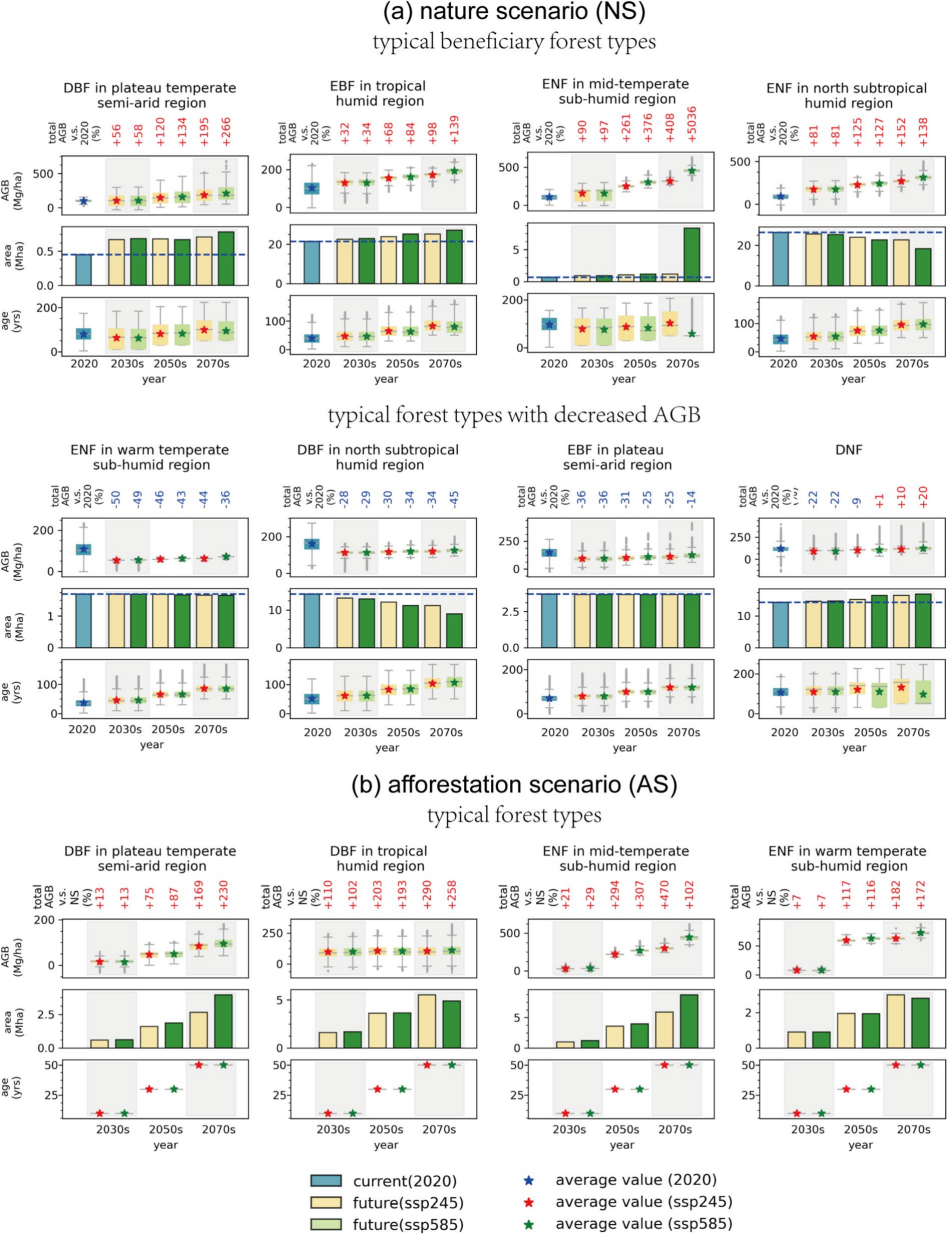
Variations of average AGB (Mg/ha), forest area (Mha), stand age (yrs) for **a** typical forest types under the nature scenario and **b** the afforestation scenario. The numbers above the x-axis indicate the total AGB variations (%) compared to the baseline year 2020, while red color represents positive variations and blue represents negative

Climate-induced changes in forest composition significantly contribute to variations in total AGB. Figure 4 displays the average AGB, forest area, stand age, and changes in total AGB across several typical forest types, with data for all 15 forest types shown in Figure S14-S15. By 2080, over 80% of forest types are projected to experience increases in total AGB. Forest types such as ENF in the mid-temperate sub-humid region and DBF in the plateau temperate semi-arid region show significant population expansions, leading to substantial carbon sequestration, particularly under the SSP585 scenario, with increases of 5036% and 266% in total AGB compared to 2020 levels, respectively. ENF in the north subtropical humid region exhibits steady growth in total AGB, with its average stand age rising from 35.83 ± 15.54 years in 2020 to 94.77 ± 25.36 to 96.56 ± 27.29 years in the 2070s. Conversely, DBF in north subtropical humid region shows a decrease in total AGB, primarily due to the severe forest area loss with an estimated loss proportion of 14.96–37.02% in the 2070s. Although similar trends are observed across most forest types under different climate scenarios, notable differences exist. For instance, ENF in the mid-temperate sub-humid region undergoes more pronounced population expansion under SSP585 compared to SSP245, while DBF in the warm/mid-temperate region experiences severe forest land loss under SSP585. These phenomena suggest that significant forest succession differences contribute to AGB variations in the north subtropical humid and mid-temperate sub-humid regions across different climate change scenarios.

### Prediction of future forest carbon sink under the afforestation scenario

Based on China’s future afforestation targets, we observed that the projected forest area increases at an approximately constant rate over time. Therefore, a first-order linear regression model is applied (R2 = 0.98, RMSE = 2.58; shown in Figure S12) to capture this trend. Using the fitted model, we interpolat that China’s forest area is expected to reach 197.71, 238.01, and 278.32 Mha by the 2030 s, 2050 s, and 2070 s, respectively. By comparing these targets with the remaining forest areas derived from the nature scenario, we estimated the additional afforestation area required for each climate scenario, as shown in Table 3.

**Table 3:** Variations of afforestation target, area, total AGB, stand age and average AGB in the afforestation scenario

Period	Climate scenario	Forest area target (Mha)	Afforestation area (Mha)	Total AGB (PgC)	Stand age (yrs)	Average AGB (Mg/ha)
2030s	SSP245	197.71	33.83	15.92 ± 5.53	55.99 ± 37.28	160.62 ± 55.79
	SSP585		34.07	16.03 ± 5.62	55.80 ± 37.39	161.78 ± 56.72
2050s	SSP245	238.01	75.55	22.80 ± 8.12	67.37 ± 38.35	191.36 ± 68.14
	SSP585		77.66	23.82 ± 8.85	66.29 ± 38.57	199.94 ± 74.32
2070s	SSP245	278.32	117.90	29.54 ± 11.98	81.03 ± 37.85	212.29 ± 86.00
	SSP585		129.32	29.72 ± 13.38	70.24 ± 32.86	213.81 ± 96.22

The newly established forests under the afforestation scenario are projected to supply cumulative forest carbon sinks. The total AGB is estimated to be 15.92 ± 5.53 PgC, 22.80 ± 8.12 PgC, and 29.54 ± 11.98 PgC in the 2030 s, 2050 s, and 2070 s under the SSP245 scenario, respectively. In comparison, under the SSP585 scenario, the estimates are 16.03 ± 5.62 PgC, 23.82 ± 8.85 PgC, and 29.72 ± 13.38 PgC for the same decades. It is noteworthy that the AGB sequestration induced by newly planted forests constitutes approximately 37.42–65.60% of the carbon accumulation achieved by existing forests in the 2070s. These newly planted forests help to offset carbon losses associated with forest degradation in the nature scenario and significantly mitigate risks, particularly under the high-emission SSP585 scenario.

Due to the young age structure and expanding area of newly planted forests, the carbon sequestration rates are projected to be higher than existing forests. Under the SSP245 scenario, these rates are estimated to be 199.91 ± 34.25, 157.66 ± 28.93, and 151.19 ± 28.85 TgC/year for the 2030 s, 2050 s, and 2070 s, respectively. Meanwhile, under the SSP585 scenario, the projected rates are 203.49 ± 35.03, 170.46 ± 32.56, and 186.61 ± 37.61 TgC/year for the same time periods.

**Fig. 5:**
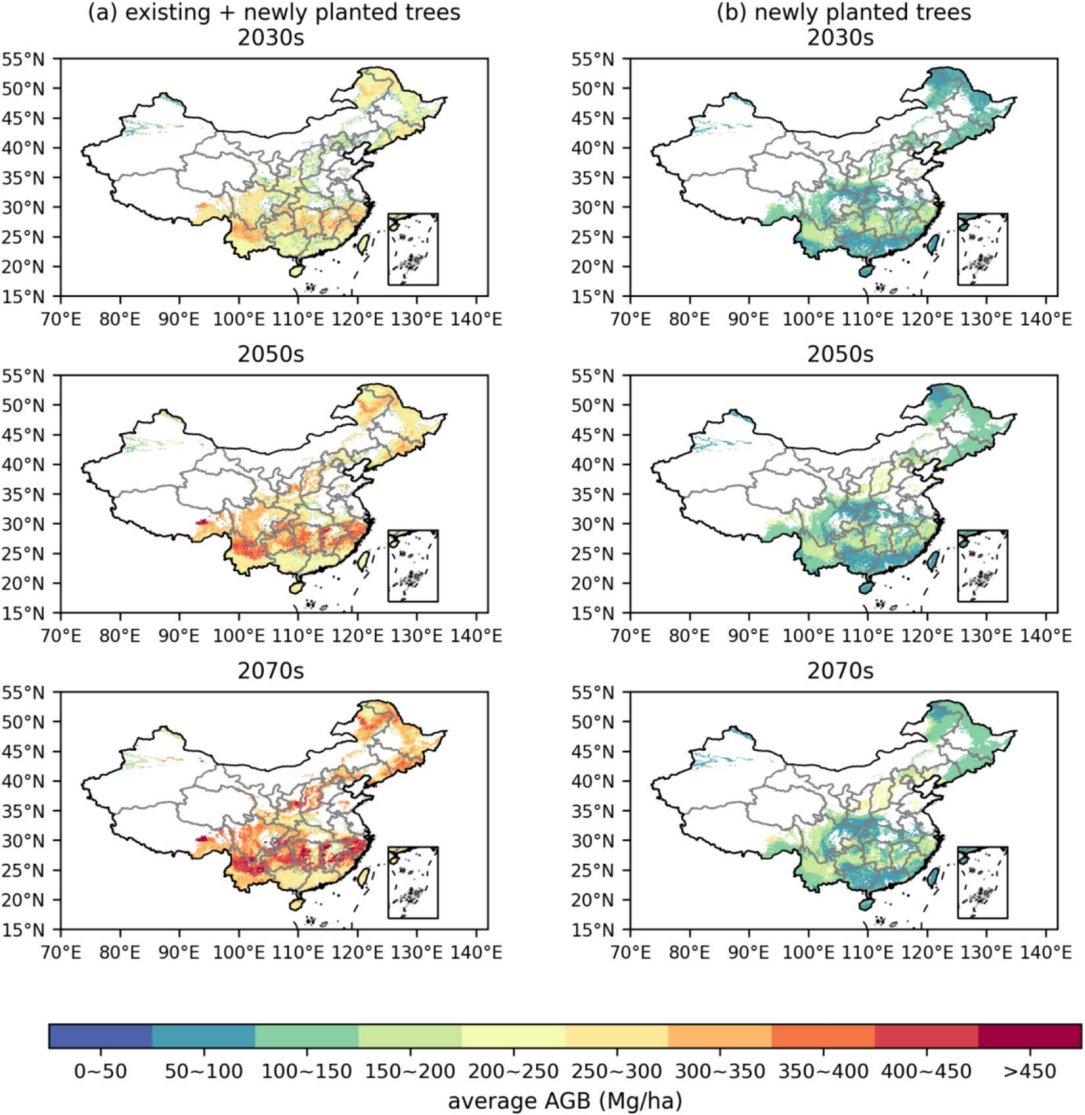
Spatial distributions of AGB (Mg/ha) of **a** existing and newly planted forests and **b** only newly planted forests under the SSP245 scenario under the afforestation scenario. All maps are averaged at a spatial resolution of a 0.1°

Figure 5 illustrates the spatial distributions of AGB under the afforestation scenario for the SSP245 scenario. The AGB distribution pattern under the SSP585 scenario is similar, as shown in Figure S16. Variations in average AGB distributions are primarily influenced by the carbon sequestration capacities of different forest types. In the north subtropical humid region, ENF exhibits rapid carbon accumulation, with an AGB density estimated at 217.40 ± 25.12 to 253.45 ± 35.10 Mg/ha in the 2070s. This results in a high AGB density zone (> 300 Mg/ha) across this region. Conversely, although DNF and EBF in temperate/subtropical humid/sub-humid regions dominate the newly planted forest area, comprising 22.76% and 17.02% of the total area in the 2070 s under the SSP245 scenario, these forest types exhibit lower carbon sequestration efficiencies, leading to lower AGB densities in the mid-temperate sub-humid region, with estimates of 98.12 ± 22.29 Mg/ha and 165.79 ± 79.81 Mg/ha, respectively.

## Discussion

### Divergence of current forest carbon estimates

The carbon stock estimates presented in our study differ from those of previous assessments. For example, Qin et al. [43] employed three growth models and national forest inventory datasets to estimate carbon stocks, arriving at a figure of 7.62 ± 0.05 PgC for 2020. Qiu et al. [44] developed a method incorporating growth-related changes and estimated the AGB stock at 9.23 PgC for the same year. Xu et al. [58] predicted the total AGB dynamics by age-biomass growth models, and got an estimation of around 15 PgC in 2020. Many efforts have been made to figure out the certain forest carbon stock in China, and their results are shown in Table S6. Apparently, estimates of forest carbon in China vary significantly.

Several factors contribute to inconsistencies across different studies, including limited long-term reliable observational data, varying accounting methods, and differences in forest classifications [18, 66]. Even minor variations in methodology can lead to substantial differences in estimates. For instance, Zhao et al. [73] applied the continuous biomass expansion factor method and estimated carbon stocks at 6.56 PgC for the 2000s. In contrast, Yao et al. [61], using the same national forest inventory data, developed a semi-empirical model that incorporates forest age and climatic factors, yielding an estimate of 10.75 PgC. In this study, we estimated China’s forest carbon stock in 2020 to be 11.59 ± 4.06 PgC, which is higher than most previous estimates. Several factors may explain this discrepancy. First, due to limited data availability, we classified forests based on climatic regions and general biological characteristics rather than tree species, as adopted in other studies (e.g., [73]). This methodological difference may introduce classification-related uncertainties. Second, although we compiled a wide range of publicly available datasets and implemented strict quality control measures, the overall sample size may still be insufficient to support precise national-scale biomass estimation [57]. Additionally, in constructing the forest age map, the ages of some grid cells were extrapolated by simply adding ten years to values from the 2010 dataset. This method neglects the potential for tree mortality and subsequent regeneration during the intervening decade. Given that biomass accumulation is generally slow during early successional stages and increases substantially during mid-successional periods, this simplified age adjustment could lead to an overestimation of biomass in areas affected by recent disturbance and regrowth. To assess the reliability of our estimates, we compare our spatial distribution of AGB with the biomass product for 2019 developed by Yang et al. [60]. The comparison reveals a strong correlation, with a coefficient of r = 0.87 (*p* < 0.05), which means that although our estimate is relatively larger, it can exhibit spatial characteristics to some extent.

### Climate-induced risks to forest carbon sink in China

Apart from estimates of current carbon stocks, predictions of future carbon sequestration rates are fraught with uncertainty. For example, Chen et al. [10] projected future carbon sinks of existing forests by extending the age-biomass growth model, resulting in an estimate of an additional 8.67 ± 6.93 PgC over the next two centuries. However, this approach overlooked crucial factors such as climate impacts and forest management strategies. To address these gaps, our study examines two scenarios: the nature scenario and the afforestation scenario.

In the nature scenario, we integrate climate variables (MAP, MAT) into our biomass-age growth models and assess the carbon sink enhancement due to CO_2_ fertilization. This scenario also accounts for the impacts of climate change on tree species selection, a factor often overlooked in prior studies [66]. Under this scenario, we map future forest distributions based on projected suitable habitat changes derived from MaxEnt models, with detailed information presented in Figure S17. Our results reveal significant differences in climate-induced changes to suitable habitats, even among species within the same group (e.g. EBF group). For example, suitable habitats of EBF in plateau semi-arid region and tropical humid region are expected to expand in the future, supporting the findings of Lei et al. [26], which indicate that climate change will lead to an expansion of potential suitable habitats for EBF in the southeastern and southern parts of the Qinghai-Tibetan Plateau. Conversely, EBF in temperate and subtropical humid/sub-humid regions is projected to experience a decline in suitable habitats. Similarly, Zhang et al. [69, 71] reported a contraction of suitable habitats for *Cyclobalanopsis glauca (Thunberg) Oersted*, a dominant tree specie in the broadleaf forests of China, in Jiangxi and Fujian Provinces, which are located in the northern subtropical humid region.

To gain a deeper understanding of the factors driving changes in suitable habitat distribution, we analyze the relative importance of predictive indicators in modeling suitable habitats, as illustrated in Figure S18. In sub-humid, sub-arid and arid regions, water-related factors, such as Precipitation of Driest Month (P_dm_) and MAP, predominantly influence the suitable habitat distribution across all forest types. In contrast, while P_dm_ remains significant in humid regions, energy-related factors, including MAT, Mean Diurnal Range (MDR), Isothemality (ISO), are also of great importance. This phenomenon can be explained by vegetation physiology. In arid or sub-arid regions, plants typically constrict the stomatal apertures on their leaves to conserve moisture when soil moisture is low, resulting in reduced transpiration and photosynthesis, which ultimately affects plant growth. Conversely, in humid regions, where soil moisture levels are higher, plants are less"water stressed", leading to the conclusion that plant growth becomes less sensitive to changes in soil moisture and is instead more influenced by energy availability [25]. The prominence of water-related factors identified in our study underscores the increasing risks associated with water availability for forest growth. As noted by Jiao et al. [24], water constraints on vegetation growth have intensified over the past three decades, with significant expansions observed in areas experiencing vegetation water deficits. Additionally, our study highlights the critical role of elevation in the plateau temperate sub-arid region (shown in Figure S18), which is particularly relevant given that the Qinghai-Tibet Plateau is characterized by a pronounced elevation gradient closely linked to vegetation growth [19, 72].

In the 2070 s, significant shifts from DBF to ENF are projected in the Greater Xing’an Mountains (located in the mid-temperate sub-humid region) under the SSP585 scenario. This transition is anticipated to result in an abnormal decrease in AGB in the region. Our analysis seeks to elucidate the underlying causes of this phenomenon. The Greater Xing’an Mountains region is expected to experience increased temperature, with a decrease in temperature variance across different time scales (as shown in Figure S4, S5, S8, S9 and S11). Notably, when summer temperatures exceed the optimal range for vegetation productivity, as identified by Zhang et al. [69, 71], it becomes evident that the mid-temperate sub-humid region will encounter these conditions earlier under the SSP585 scenario compared to the SSP245 scenario. This trend indicates a heightened risk of heat stress, particularly in the SSP585 scenario. The decline of DBF in warm and mid-temperate regions, whose suitable habitat is closely associated with P_dm_ (the relative importance, hereafter, 42.93%), MDR (13.90%), MAT (12.23%), ISO (9.52%) and elevation (7.88%), is likely attributable to consistently high temperatures. Previous studies [21] have demonstrated that rising temperatures accelerate the reduction in radial growth and contribute to the mortality of *Populus davidiana*, a typical DBF species in this region. Conversely, the distribution of suitable habitat for ENF in the mid-temperate sub-humid region is predominantly determined by P_dm_ (86.39%) and is less influenced by energy-related factors. This finding contrasts with some research suggesting that temperature is a significant determinant of ENF growth [45]. We derive the forest habitat suitability distribution using the MaxEnt model, which is known to be sensitive to sampling bias and susceptible to overfitting, potentially leading to inaccuracies in model results [27, 40]. Consequently, there is a possibility that we may have overestimated the AGB sequestration capacity of ENF in the mid-temperate sub-humid region. Despite these uncertainties, our findings align with previous studies indicating that climate-induced risks pose a significant threat to forest carbon sinks in China [63].

### Important role of afforestation in forest carbon sink sequestration

In the afforestation scenario, we project the future suitability of habitats for various forest types and adopt a"most suitable tree"strategy. Similar efforts have been made to evaluate the importance of afforestation, and different management strategies yield varying estimates. For example, Qin et al. [43] proposed that eucalyptus (Eucalyptus spp.) and poplar (Populus spp.) plantations will be replaced by broad-leaved or coniferous-broadleaf mixed forests, predicting that this species replacement approach could enhance carbon sinks by 0.09 PgC/yr by 2030 and 0.06 PgC/yr by 2060. Xu et al. [58] focused on selecting species for maximal carbon stock on potential afforestation sites, estimating that such an approach could sustain a biophysical carbon sink of 0.4 PgC/yr by 2060 and 0.2 PgC/yr by 2100. Despite disparities in existing estimates, there is partial consensus that China’s current forest carbon sink will diminish in the near future [48]. Our study underscores the potential benefits of afforestation in bolstering forest carbon sequestration.

However, it is important to note that the carbon sequestration rate of afforestation should not be overestimated, particularly in long-term projections. Our findings indicate that 60% of forest types are likely to experience decline in suitable habitats in the future (as illustrated in Figure S17). This suggests that the carbon sink capacity of newly planted forests will decrease once afforestation opportunities are exhausted. Notably, a higher risk of habitat loss is observed under high-emission scenarios. Therefore, in face of climate change, there is an urgent need to reduce fossil fuel emissions and implement other mitigation strategies [22].

### Potential afforestation policy implications

China has launched several large-scale forest policy initiatives in recent decades, such as the Natural Forest Conservation Program and the Grain for Green Project. While these efforts have substantially expanded the area of afforestation, long-term effectiveness remains limited. For example, the average tree survival rate in afforestation projects implemented between 1952 and 2005 was only 24% [55], with survival rates as low as 15% in the Three-North Shelter Forest System Project [7]. A key shortcoming of current afforestation policy lies in its insufficient alignment with local environmental conditions. The widespread use of inappropriate species and the overemphasis on tree and shrub planting, without adequate ecological considerations, have hindered the achievement of long-term environmental and policy objectives [8].

In this study, we identified suitable forest types for potential afforestation by evaluating their ecological feasibility under current local conditions, as documented in the national forest inventory. This approach is intended to reduce the risk of afforestation failure and enhance long-term carbon sequestration outcomes. The afforestation suitability map we produced can serve as a valuable reference for policymakers seeking to improve afforestation strategies through better ecological matching and targeted implementation. In addition, our nature scenario provides projections of forest dynamics under future climate change, offering valuable insights for identifying climate-vulnerable regions and informing the development of targeted forest conservation strategies.

### Limitations of the afforestation scenario and future research direction

Although this study provides valuable insights, it is important to recognize that our projections represent an idealized afforestation scenario. First, our analysis does not account for natural disturbances or other destructive factors. Emerging evidence indicates that climate change is intensifying the frequency and severity of extreme events, including temperature extremes [56], heat stress [3], and droughts [68], all of which can negatively impact forest growth and carbon sequestration potential.

Second, the afforestation map used in this study is based solely on biophysical forest suitability, without incorporating socio-economic and policy constraints. In reality, afforestation feasibility is also shaped by institutional and economic factors. For instance, China’s national zoning program for ecological functions divides the country into three major ecological zones and 48 subzones based on climatic and ecological characteristics [37]. In water conservation zones, large-scale afforestation is strictly limited, while in agricultural production zones, farmland is protected to maintain the minimum arable land threshold of 120 Mha mandated by the government. In urban cluster zones, financial incentives and government policies can significantly affect local livelihoods [53], thereby influencing land-use decisions and afforestation efforts [62].

Therefore, our findings should be interpreted as an idealized estimate rather than a fully practical or implementable afforestation strategy. They highlight the potential role of afforestation in mitigating climate-related risks, while underscoring the necessity for more comprehensive and context-sensitive planning. To develop a more realistic future afforestation scenario, further efforts are required. First, improved methods are needed to generate more accurate forest suitability maps that account not only for biophysical growth conditions but also for natural disturbances and forest management policies. Second, more details should be incorporated into afforestation planning, including updated policy developments, implementation costs, and socio-economic constraints.

## Conclsions

In this study, we develop methods that account for forest age, climatic factors (MAP and MAT), and CO_2_ fertilization effects to estimate the forest biomass stock at 11.59 ± 4.06 Pg C for the year 2020. Furthermore, we employ various forest management and climate change scenarios to predict changes in carbon storage, forest distribution, forest composition, age structure, and the carbon sink capacity of different forest types over the next 60 years.

In the nature scenario, we integrate growth models with SDMs to forecast forest dynamics. Our analysis reveals that climate change significantly affects forest suitability, leading to reductions in forest area and shifts in composition. We observe a declining trend in the carbon sequestration rate across all future climate change scenarios, indicating a diminished potential for existing forests to act as carbon sinks. Notably, severe loss of suitable land in the mid-temperate sub-humid region by the 2070 s presents a heightened risk under the high-emission scenario (SSP585), with CO_2_ fertilization effects unable to counterbalance this loss.

In the afforestation scenario, we propose an optimal strategy where the most suitable tree species are planted in potential afforestation areas. Our results suggest that these newly planted forests could contribute approximately 37.42–65.60% of the carbon sink capacity of existing forests in the 2070 s, thereby substantially mitigating climate-induced risks.

## Supplementary Information

Additional file 1.

## Data Availability

The forest survey data supporting the conclusions of this article is available in the figshare repository: https://doi.org/10.6084/m9.figshare.27130098.v1. Other data that support the findings of this study are derived from the following resources available in the public domain: GLC_FCS30 forest cover data is free access at https://doi.org/10.5281/zenodo.3986872. ESA CCI LC data is openly available at https://maps.elie.ucl.ac.be/CCI/viewer/download.php. The forest age map from Cheng et al. [11] can be downloaded at https://doi.org/10.5281/zenodo.8354262. The forest age map developed by Shang et al. [48] is available at https://doi.org/10.6084/m9.figshare.24464170.v1. The MPI-BGC global forest age map from Besnard et al. [4] is available at https://doi.org/10.5194/essd-13-4881-2021. The climate and bioclimatic data in the current and future periods are derived from WorldClim (http://www.worldclim.orghttp://www.worldclim.org). The 1pt CO_2_ experiment results of CMIP6 are downloaded from https://esgf-node.llnl.gov/search/cmip6/. The elevation data is downloaded from the ETOPO Global Relief Model (version: ETOPO 2022) (https://www.ncei.noaa.gov/products/etopo-global-relief-model), while the slope variable is calculated based on the elevation in Arcgis 10.8.1. All the soil variables are obtained from the Harmonized World Soil Database (HWSD v 1.21) (http://www.iiasa.ac.at/web/home/research/researchPrograms/water/HWSD.html).

## Abbreviations

AGB: Aboveground biomass
SDM: Species distribution model
MAT: Mean annual temperature
MAP: Mean annual precipitation
ENF: Evergreen needle-leaved forest
DNF: Deciduous needle-leaved forest
EBF: Evergreen broad-leaved forest
DBF: Deciduous broad-leaved forest
MF: Mixed forest
MM: Michaelis–Menten growth model
MO: Monomolecular growth model
L: Logistic growth model
NEP: Net ecosystem productivity
RMSE: The root mean square error
R^2^: The coefficient of determination
AIC: Akaike information criterion
GCM: General circulation model
CMIP6: The coupled model intercomparison project phase 6
2030s: Periods of 2021–2040
2050s: Periods of 2041–2060
2070s: Periods of 2061–2080
AUC: The area under the curve
ROC: Receiver operating characteristic
MTSS: Maximum training sensitivity plus specificity
P: Suitability probability
P_dm_: Precipitation of Driest month
MDR: Mean Diurnal range
ISO: Isothemality
