# Supplementary material for: Afforestation as a mitigation strategy: countering climate-induced risk of forest carbon sink in China
Source: Carbon Balance Manag. 2025 Jun 21;20:18. doi: 10.1186/s13021-025-00308-1 (PMC12182680; doi:10.1186/s13021-025-00308-1)
Supplement: Supplementary file 1 — Additional file 1. [file 13021_2025_308_MOESM1_ESM.docx]

**supplementary materials**

**Afforestation as a Mitigation Strategy: Countering Climate-induced Risk of Forest Carbon Sink in China**

Yuan Cao^a,b,c^, Deyu Zhong^a,b,c^, Rong Shang^d^, Qihua Ke^a,b,c^, Mingxi Zhang^a,b,c^, Di Xie^a,b,c^, Shutong Liu^a,b,c^, Chensong Zhao^a,b,c^,Randongfang Wei^a,b,c^

a State Key Laboratory of Hydroscience and Engineering ,Tsinghua University, Beijing, 100084

b Key Laboratory of Hydrosphere Sciences of the Ministry of Water Resources, Tsinghua University, Beijing,100084

c Department of Hydraulic Engineering, Tsinghua University, Beijing,100084

d Key Laboratory of Humid Subtropical Eco-Geographical Process of Ministry of Education, School of Geographical Sciences, Fujian Normal University, Fuzhou, 350117

**Correspondence:** Deyu Zhong

**Email:** [zhongdy@mail.tsinghua.edu.cn](mailto:zhongdy@mail.tsinghua.edu.cn)

**Tel:** +86-13601274636

**Fax:** 010-62788544

**This file includes:**

Figures: S1-S18

Tables:S1-S6

# Figure

#
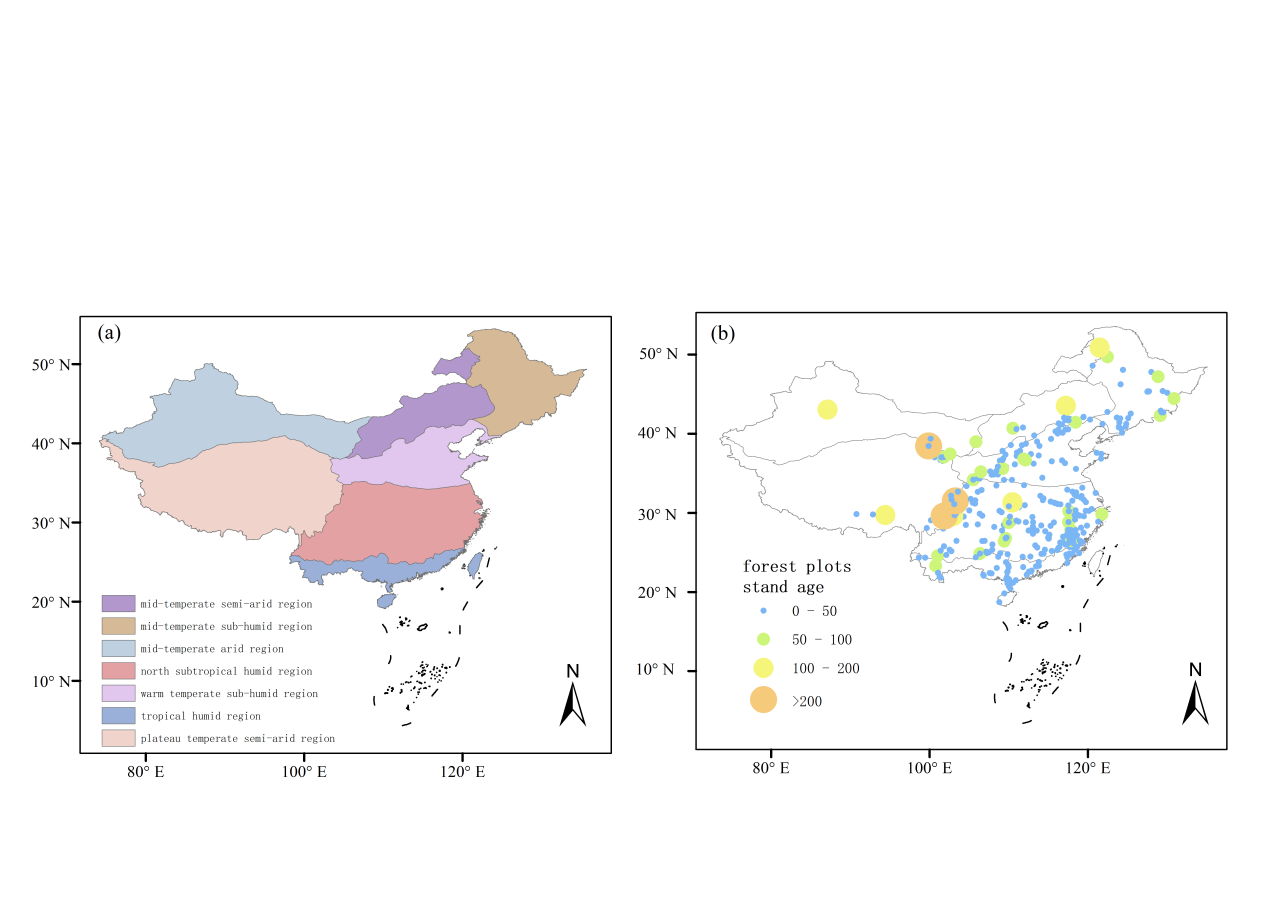


**Figure S1** (a) Division of 7 climatic zones in China and (b) spatial distribution of our forest field survey data points.

**
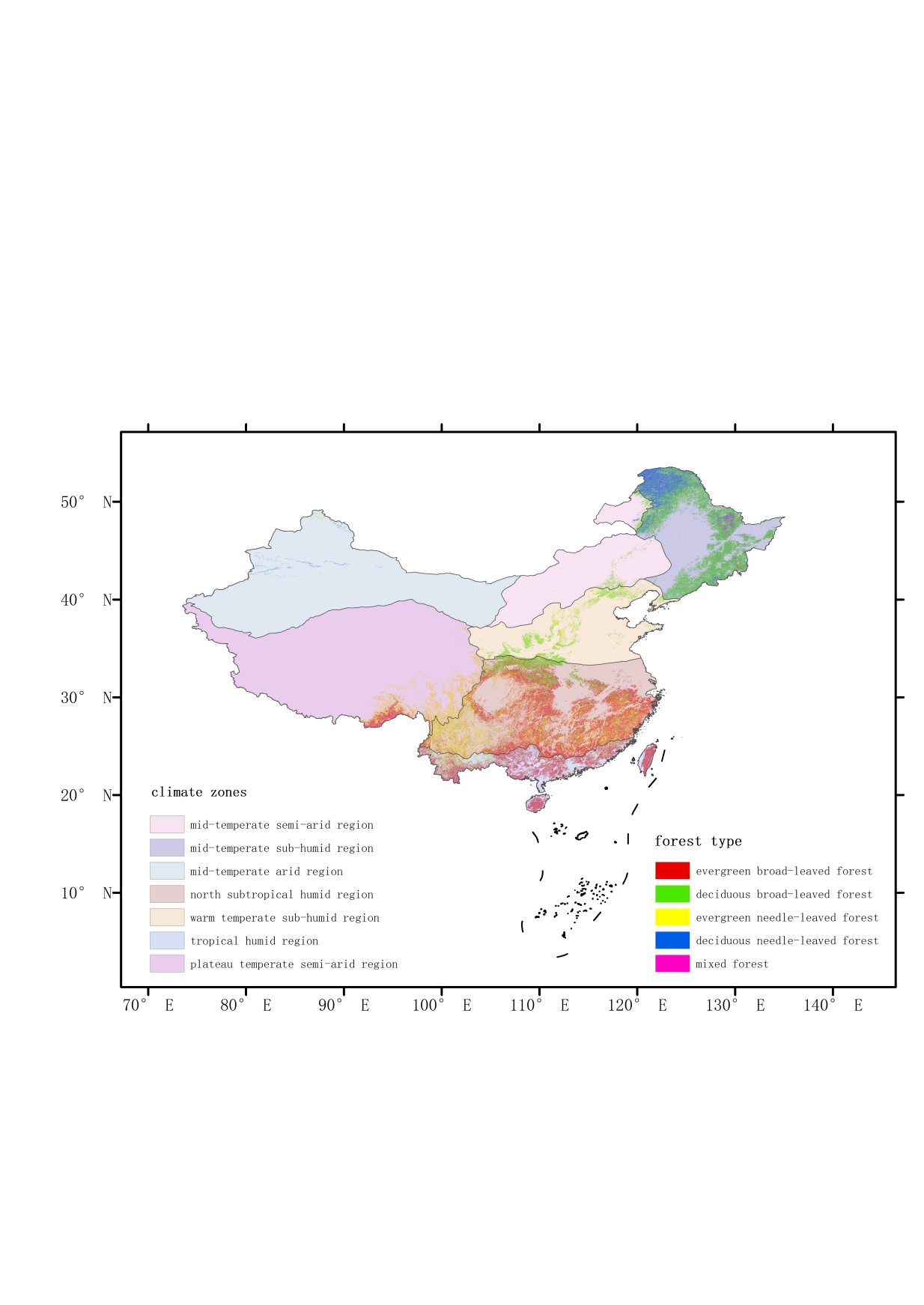
**

**Figure S2** Spatial distributions of forest cover type used in this study (on a 30 arc-second solution).


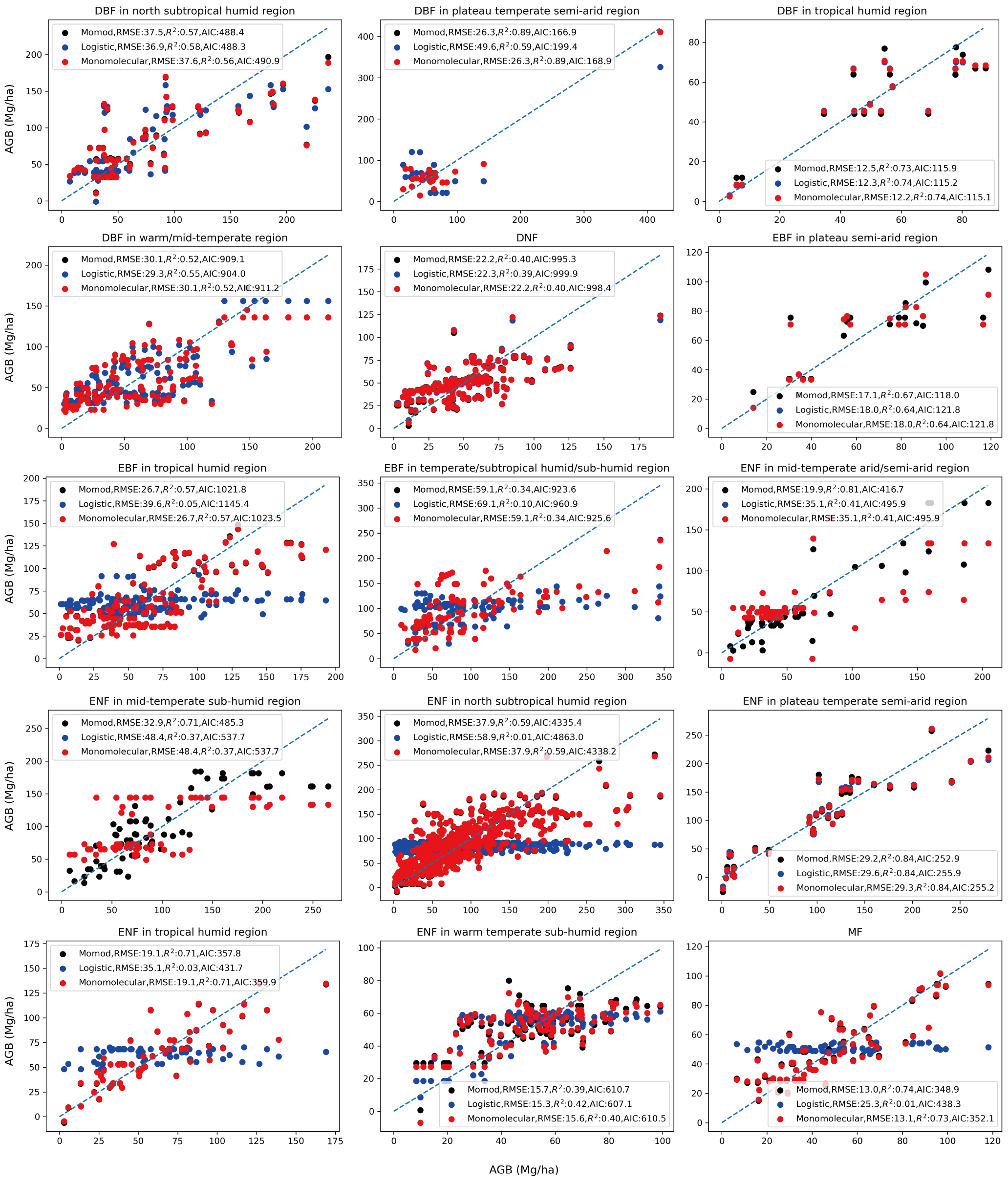


**Figure S3** Comparison between observed values (x, abscissa) and modeled values (y, ordinate) fitted by 3 growth models. The blue dotted line indicates y=x.

**Figure S4** Future distribution of Temperature Seasonality (TS).(a) shows the difference between future and baseline year 2020 (SSP245 scenario minus baseline year 2020), and (b) shows the difference between SSP585 and SSP245 scenario (SSP585 minus SSP245). Note that the Nansha Islands are not on the map due to lack of data.

**Figure S5** Same as Figure S4, but for Max Temperature of Warmest Month (T_max_, ℃)

**Figure S6** Same as Figure S4, but for Precipitation Seasonality (Coefficient of Variation, P_s_, mm).


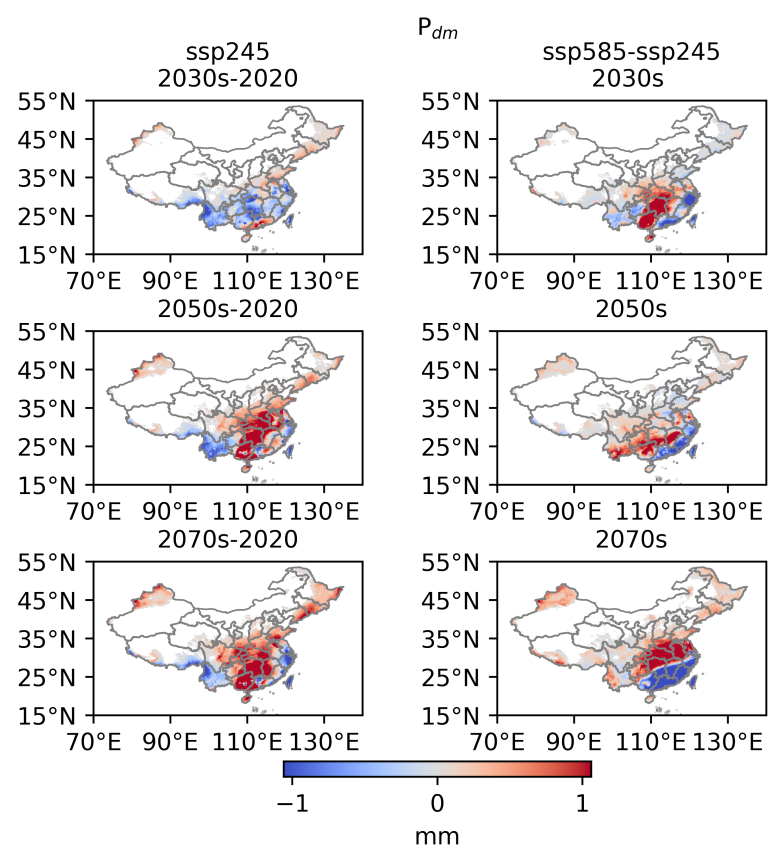


**Figure S7** Same as Figure S4, but for Precipitation of Driest Month (P_dm_, mm).


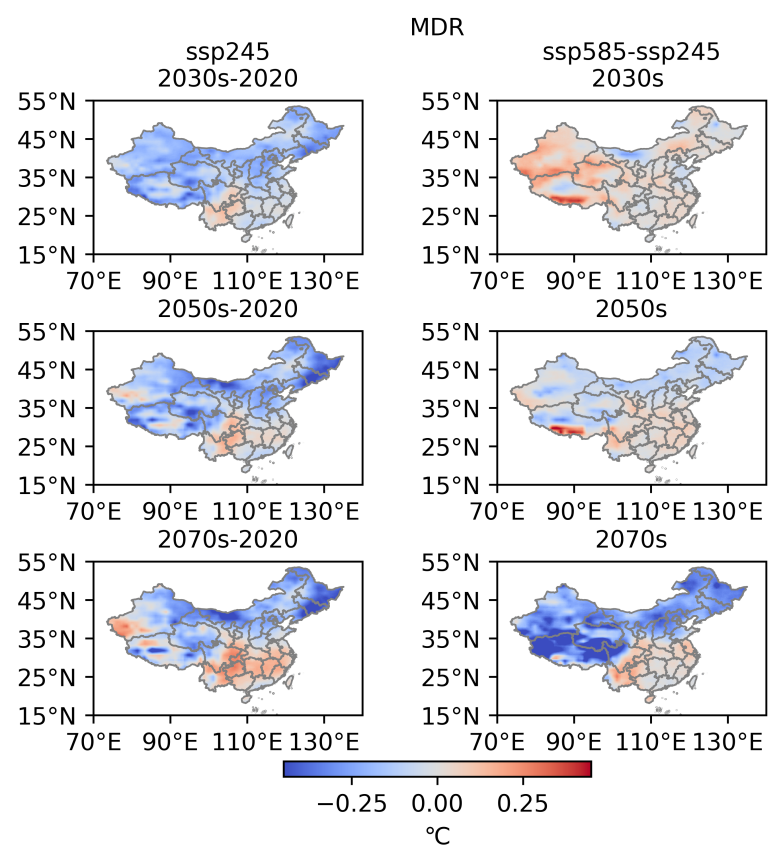


**Figure S8** Same as Figure S4, but for Mean Diurnal Range (MDR, ℃).


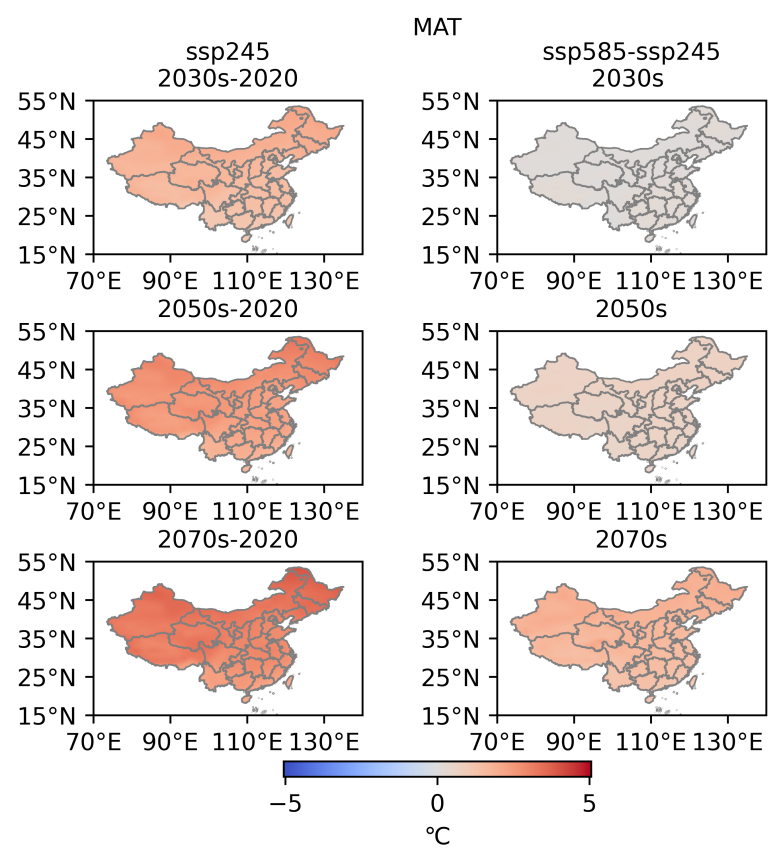


**Figure S9** Same as Figure S4, but for Annual Mean Temperature (MAT, ℃).


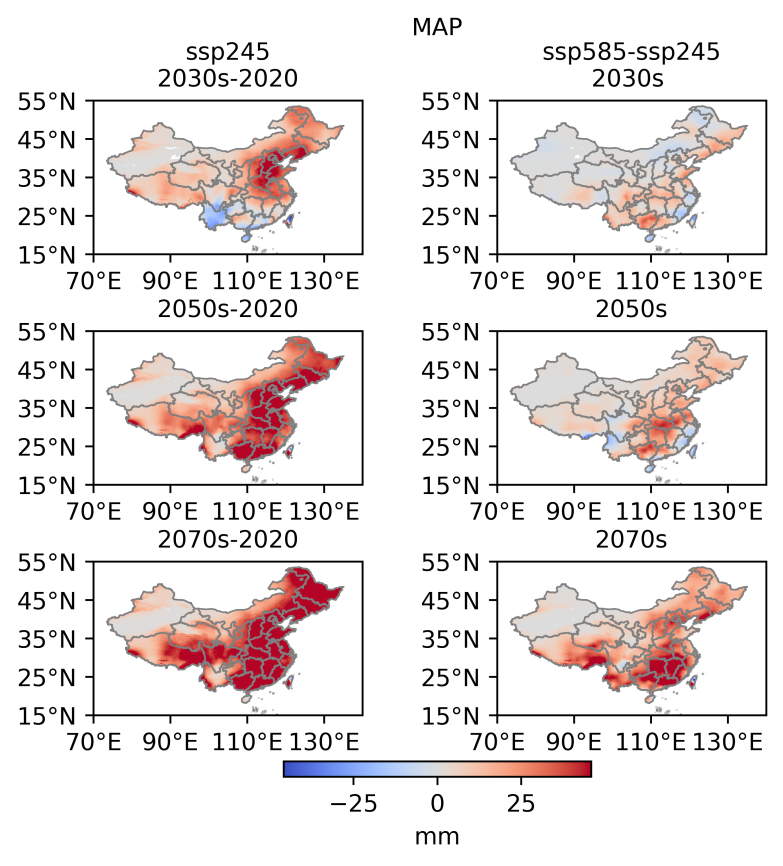


**Figure S10** Same as Figure S4, but for Annual Mean Precipitation (MAP, mm).


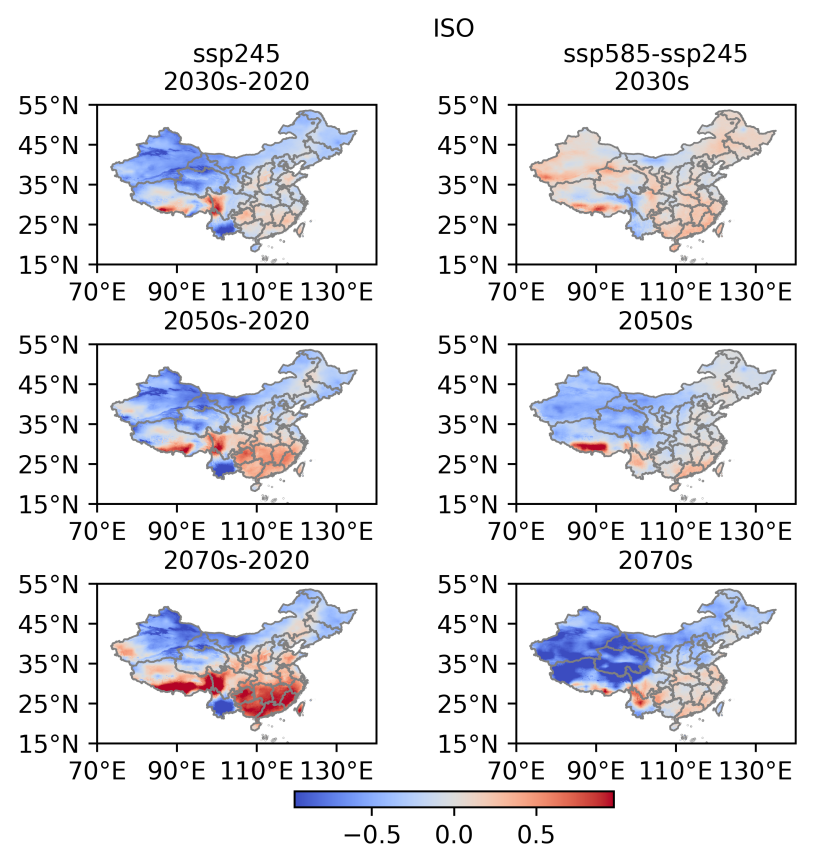


**Figure S11** Same as Figure S4, but for Isothermality (ISO).


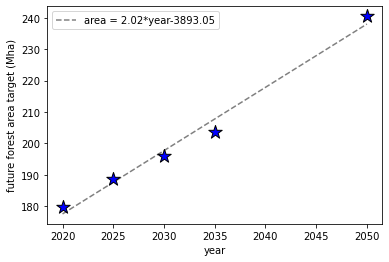


**Figure S12** Future national targets and the liner regression result (R^2^=0.98, RMSE=2.58).


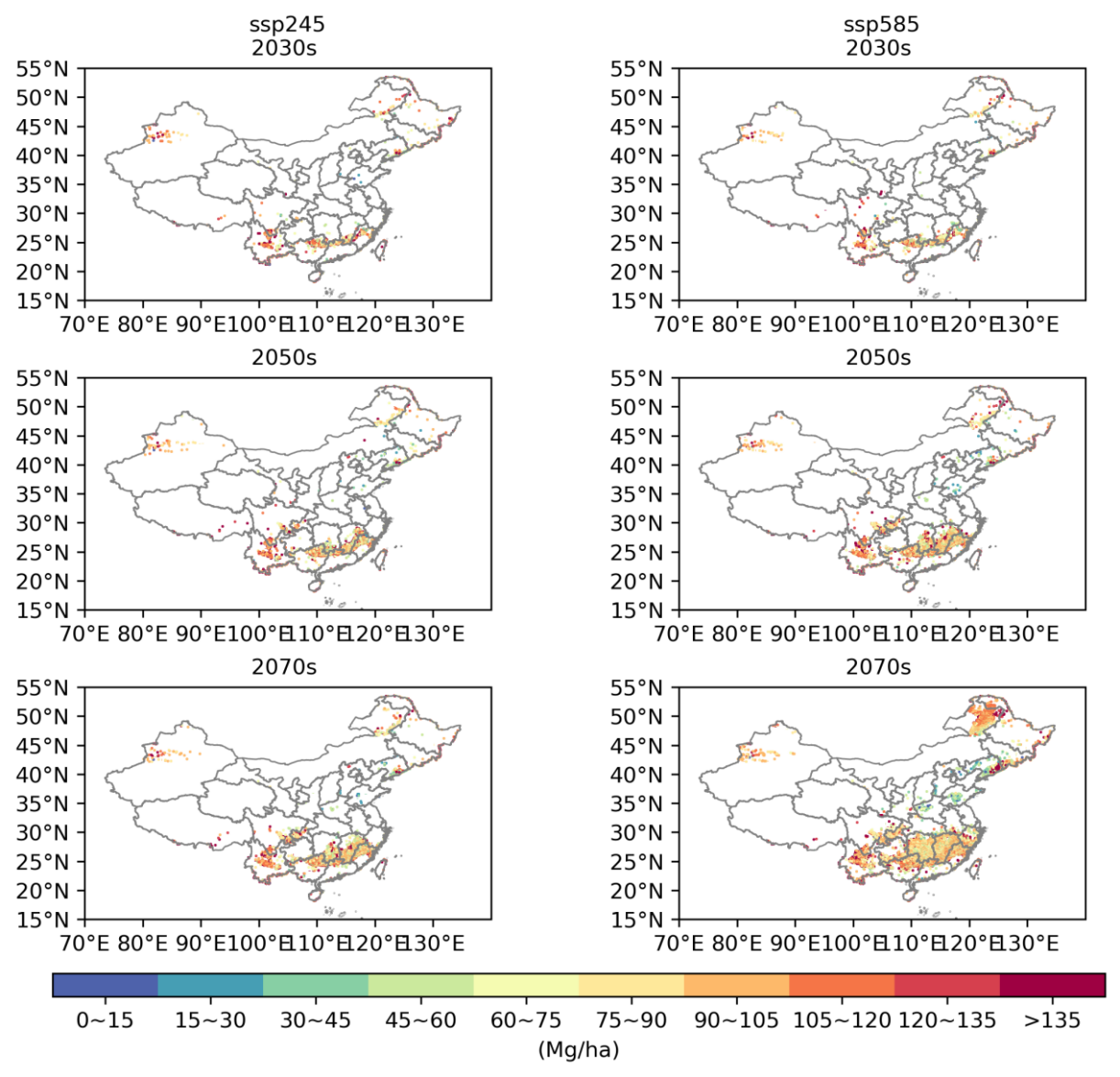


**Figure S13** Distributions of loss forest area under (a) the SSP245 scenario and (b) the SSP585 scenario, the color indicate the average AGB of loss area (Mg/ha). Note that the Nansha Islands are not on the map due to lack of data.


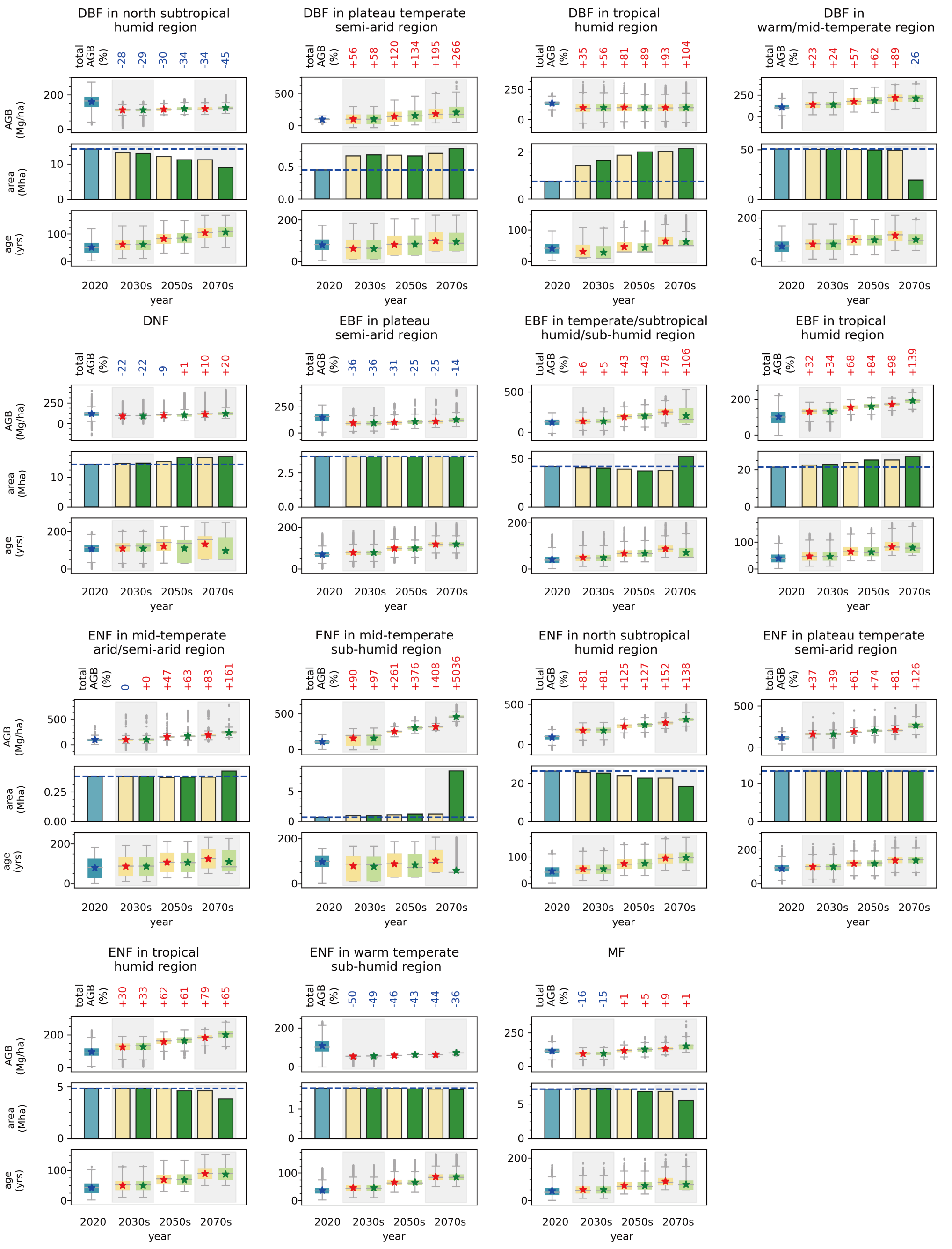


**Figure S14** Same with Figure 4 but for all forest types under the nature scenario.


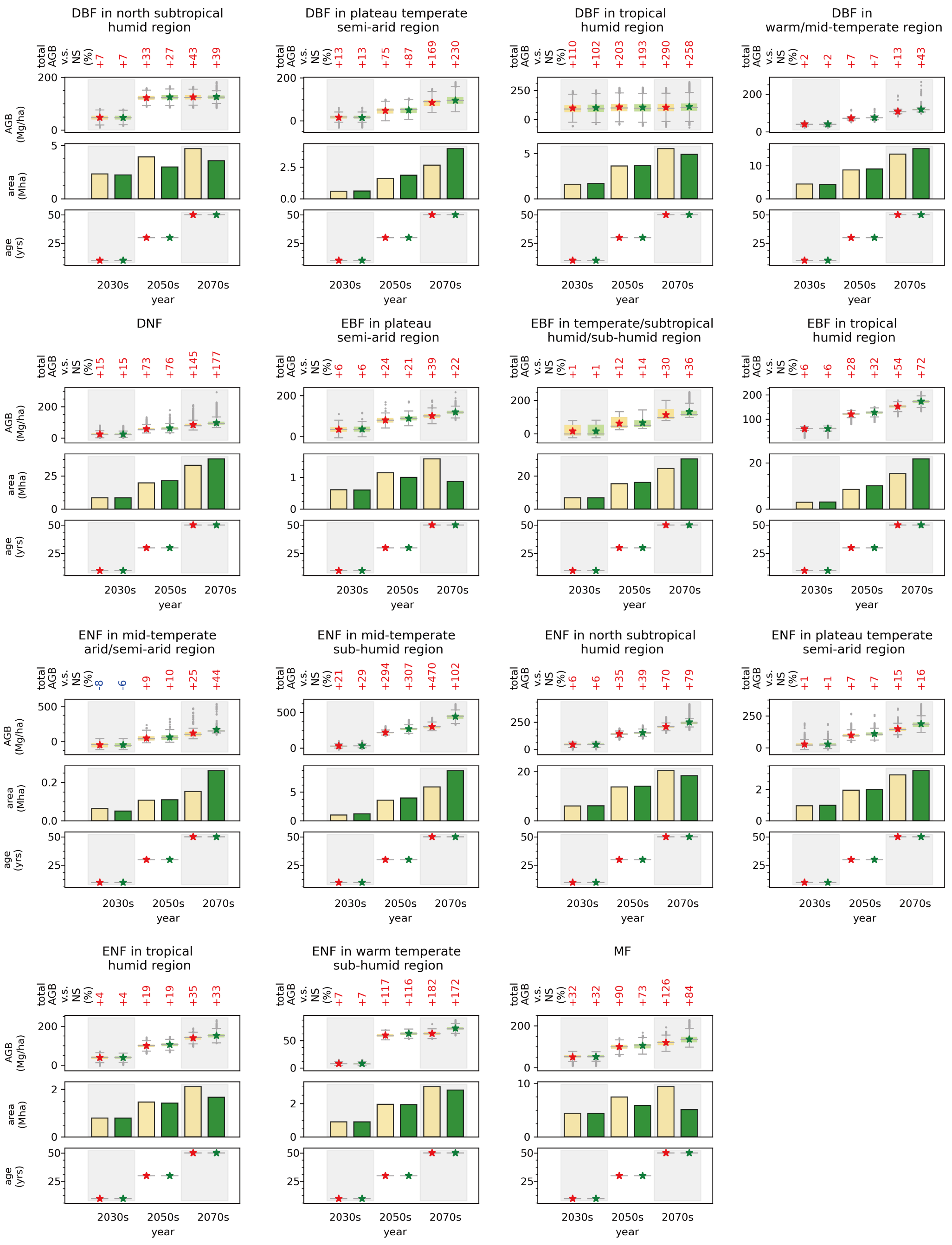


**Figure S15** Same with Figure 4 but for all forest types under the afforestation scenario.


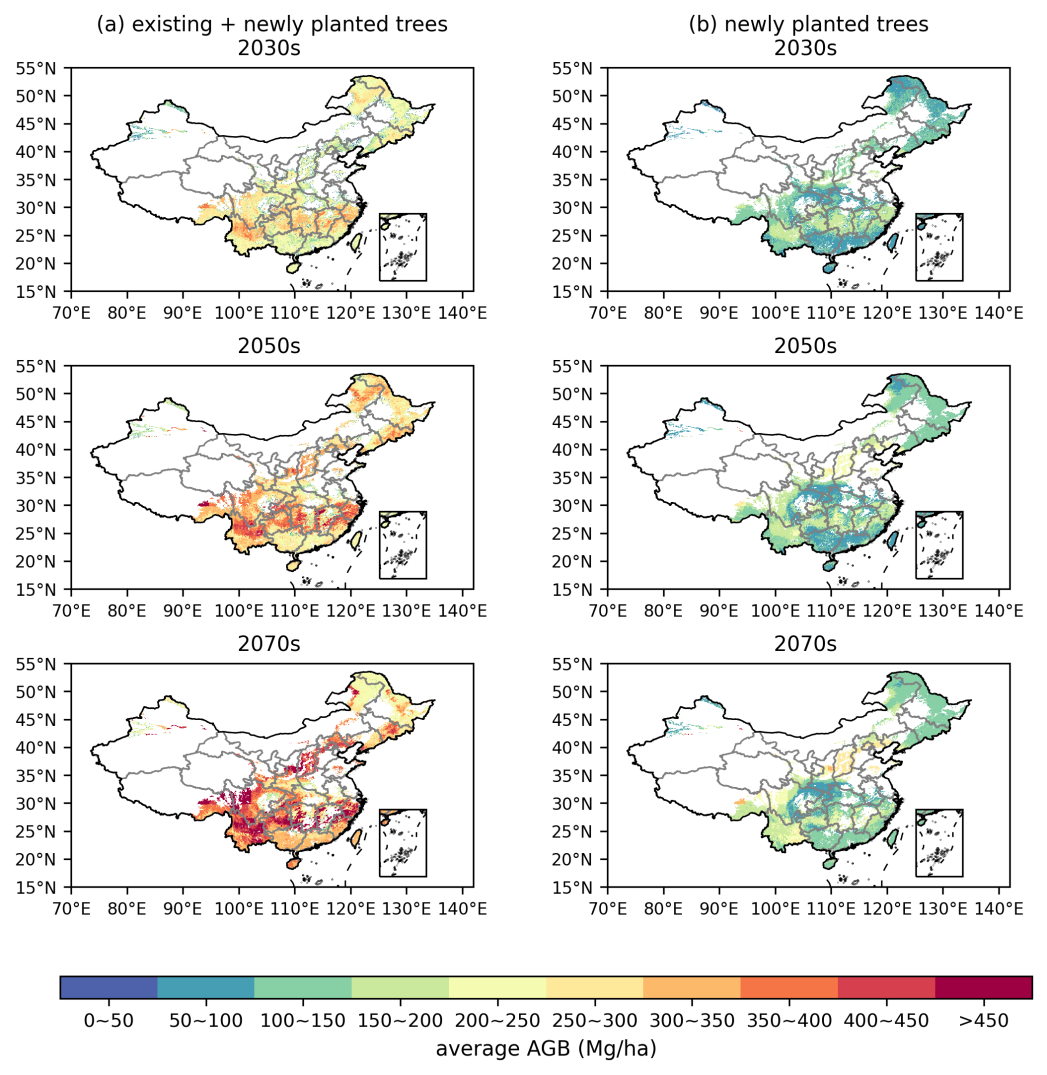


**Figure S16** Same with Figure5, but for the SSP585 scenario.


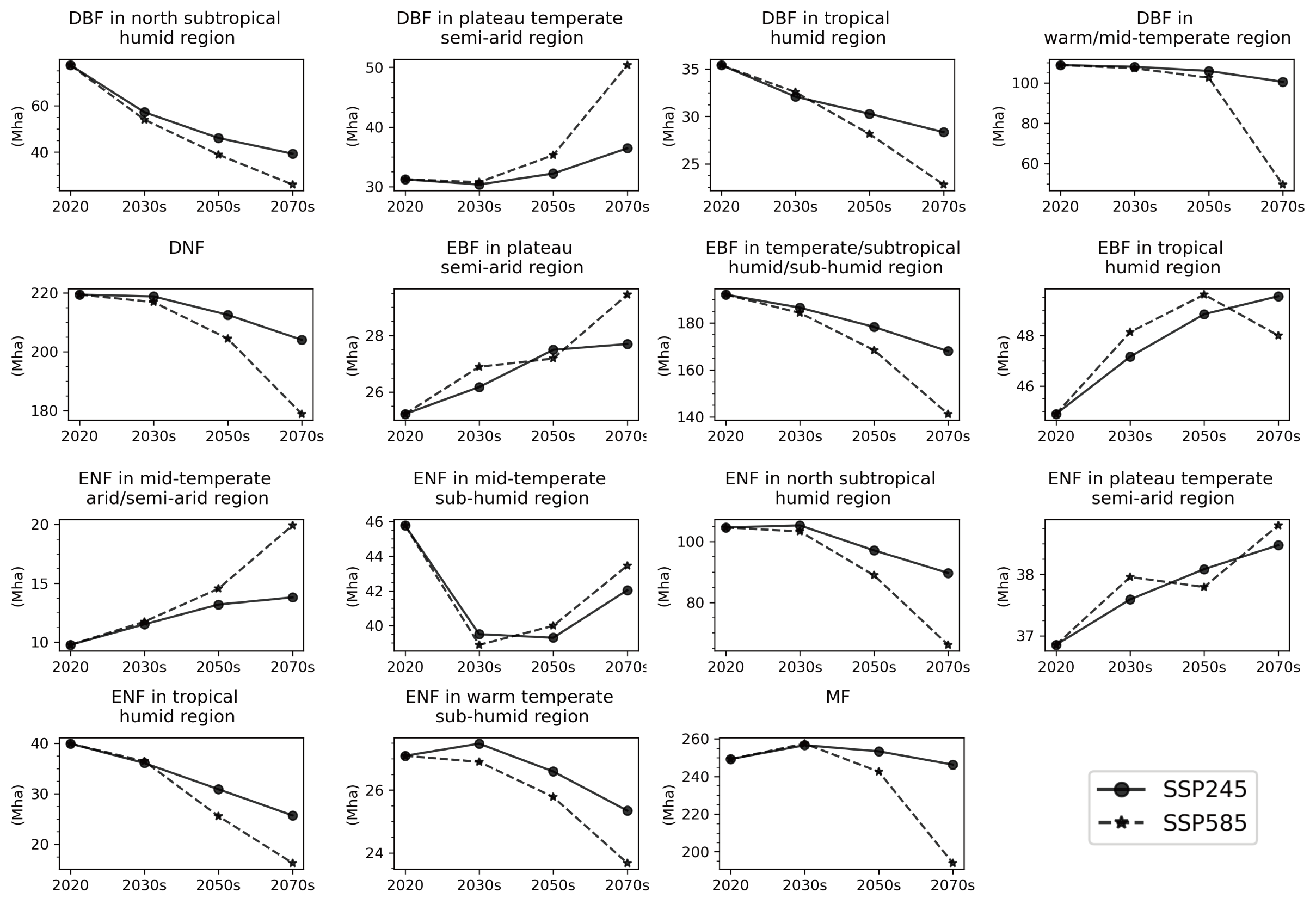


**Figure S17** Variations of suitable habitats (P>MTSS) area.


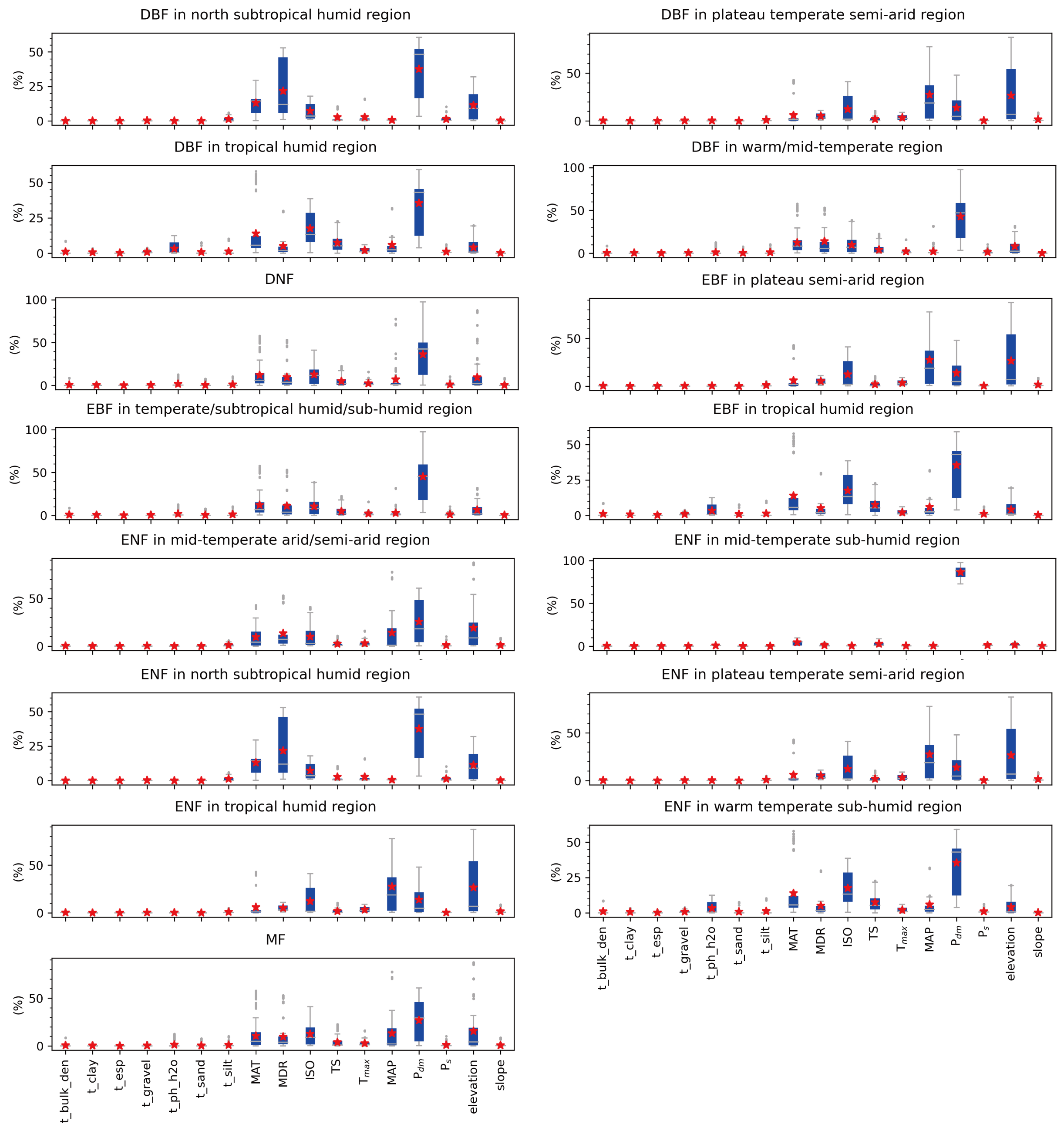


**Figure S18** Relative importance (%) in predictions of future suitable habitats of different indicators across 15 main forest types.

# Tables

**Table S1** AUC and MTSS values of different forest types.

| forest type | sub forest type | AUC | MTSS |
| --- | --- | --- | --- |
| DBF in north subtropical humid region | DBF in north subtropical humid region | 0.72 | 0.50 |
| DBF in plateau temperate semi-arid region | DBF in plateau temperate semi-arid region | 0.95 | 0.18 |
| DBF in tropical humid region | DBF in tropical humid region | 0.94 | 0.22 |
| DBF in warm/mid-temperate region | DBF in mid-temperate semi-arid region | 0.95 | 0.25 |
|  | DBF in mid-temperate arid region | 1.00 | 0.01 |
|  | DBF in warm temperate sub-humid region | 0.84 | 0.32 |
|  | DBF in mid-temperate sub-humid region | 0.65 | 0.46 |
| DNF | DNF in mid-temperate arid region | 0.98 | 0.12 |
|  | DNF in north subtropical humid region | 0.88 | 0.32 |
|  | DNF in mid-temperate semi-arid region | 0.99 | 0.12 |
|  | DNF in warm temperate sub-humid region | 0.97 | 0.11 |
|  | DNF in subtropical humid region | 0.97 | 0.11 |
|  | DNF in plateau temperate semi-arid region | 0.97 | 0.13 |
|  | DNF in mid-temperate sub-humid region | 0.78 | 0.40 |
| EBF in plateau semi-arid region | EBF in plateau semi-arid region | 0.92 | 0.20 |
| EBF in tropical humid region | EBF in tropical humid region | 0.73 | 0.45 |
| EBF in temperate/subtropical humid/sub-humid region | EBF in north subtropical humid region | 0.61 | 0.50 |
|  | EBF in warm temperate sub-humid region | 0.98 | 0.09 |
|  | EBF in mid-temperate sub-humid region | 0.93 | 0.23 |
| ENF in mid-temperate arid/semi-arid region | ENF in mid-temperate semi-arid region | 0.99 | 0.10 |
|  | ENF in mid-temperate arid region | 0.99 | 0.06 |
| ENF in mid-temperate sub-humid region | ENF in mid-temperate sub-humid region | 0.94 | 0.24 |
| ENF in north subtropical humid region | ENF in north subtropical humid region | 0.63 | 0.55 |
| ENF in plateau temperate semi-arid region | ENF in plateau temperate semi-arid region | 0.80 | 0.30 |
| ENF in tropical humid region | ENF in tropical humid region | 0.85 | 0.40 |
| ENF in warm temperate sub-humid region | ENF in warm temperate sub-humid region | 0.93 | 0.21 |
| MF | MF in mid-temperate sub-humid region | 0.94 | 0.22 |
|  | MF in north subtropical humid region | 0.76 | 0.38 |
|  | MF in warm temperate sub-humid region | 0.98 | 0.13 |
|  | MF in tropical humid region | 0.90 | 0.29 |
|  | MF in plateau temperate semi-arid region | 0.97 | 0.09 |
|  | MF in mid-temperate semi-arid region | 0.99 | 0.13 |

**Table S2** List of CMIP6 models used in this study. Detailed information can be found on the website https://pcmdi.llnl.gov/CMIP6/.

| data use | CMIP6 models |
| --- | --- |
| future climate data | ACCESS-CM2,  BCC-CSM2-MR,  CMCC-ESM2,  EC-Earth3-Veg,  FIO-ESM-2-0,  GISS-E2-1-G,  HadGEM3-GC31-LL,  INM-CM5-0,  IPSL-CM6A-LR,  MIROC6,  MPI-ESM1-2-HR,  MRI-ESM2-0,  UKESM1-0-LL |
| 1 pt CO_2_ experiment | ACCESS-ESM1-5,  BCC-CSM2-MR,  BCC-ESM1,  CESM2-WACCM,  CESM2,  CMCC-CM2-SR5,  CMCC-ESM2,  CanESM5,  MPI-ESM-1-2-HAM,  MPI-ESM1-2-LR,  SAM0-UNICON,  TaiESM1 |

**Table S3** Compare results between our map and statistical data from “Report on China's Forest Resources (2014-2018)”

| area | our map  ($\times$10^4^ ha) | statistical data from “Report on China's Forest Resources (2014-2018)”  ($\times$10^4^ ha) |
| --- | --- | --- |
| total | 1.65$\times$10^4^ | 1.7989$\times$10^4^ |
| broad-leaved forest | 10756.93 | 10447.01 |
| needle-leaved forest | 5127.506 | 5882.02 |
| mixed forest | 633.2651 | 1898.48 |

**Table S4** Variables used in MaxEnt models. State “√” means that the variable is used.

| Category | | Variable | Description | Unit | State | |
| --- | --- | --- | --- | --- | --- | --- |
| Climate | | MAT | Annual Mean Temperature | ℃ | √ | |
|  |  | MDR | Mean Diurnal Range: Mean of monthly (max temp - min temp) | ℃ | √ | |
|  |  | ISO | Isothermality |  | √ | |
|  |  | TS | Temperature Seasonality (standard deviation ×100) |  | √ | |
|  |  | Tmax | Max Temperature of Warmest Month | ℃ | √ | |
|  |  | Tmin | Min Temperature of Coldest Month | ℃ |  | |
|  |  | TAR | Temperature Annual Range | ℃ |  | |
|  |  | T_w_ | Mean Temperature of Wettest Quarter | ℃ |  | |
|  |  | T_d_ | Mean Temperature of Driest Quarter | ℃ |  | |
|  |  | T_warm_ | Mean Temperature of Warmest Quarter | ℃ |  | |
|  |  | T_cold_ | Mean Temperature of Coldest Quarter | ℃ |  | |
|  |  | MAP | Annual Precipitation | mm | √ | |
|  |  | P_wm_ | Precipitation of Wettest Month | mm |  | |
|  |  | P_dm_ | Precipitation of Driest Month | mm | √ | |
|  |  | P_s_ | Precipitation Seasonality (Coefficient of Variation) | mm | √ | |
|  |  | P_wq_ | Precipitation of Wettest Quarter | mm |  | |
|  |  | P_dm_ | Precipitation of Driest Quarter | mm |  | |
|  |  | P_warmq_ | Precipitation of Warmest Quarter | mm |  | |
|  |  | P_coldq_ | Precipitation of Coldest Quarter | mm |  | |
| Topography | | Elevation | / | m | √ | |
|  |  | Slope | / | ˚ | √ | |
| soil | t_bulk_den | Topsoil Bulk Density | kg/dm^3^ | | √ |  |
|  | s_bulk_den | Subsoil Bulk Density | kg/dm^3^ | |  |  |
|  | t_clay | Topsoil Clay Fraction | % | | √ |  |
|  | s_clay | Subsoil Clay Fraction | % | |  |  |
|  | t_gravel | Topsoil Gravel Content | % | | √ |  |
|  | s_gravel | Subsoil Gravel Content | −log(H^+^) | |  |  |
|  | t_ph_H_2_O | Topsoil pH (H_2_O) | −log(H^+^) | | √ |  |
|  | s_ph_H_2_O | Subsoil pH (H_2_O) | % | |  |  |
|  | t_esp | Topsoil Sodicity (ESP, exchangeable sodium percentage) | % | | √ |  |
|  | s_esp | Subsoil Sodicity (ESP, exchangeable sodium percentage) | % | |  |  |
|  | t_sand | Topsoil Sand Fraction | % | | √ |  |
|  | s_sand | Subsoil Sand Fraction | % | |  |  |
|  | t_silt | Topsoil Silt Fraction | % | | √ |  |
|  | s_silt | Subsoil Silt Fraction | % | |  |  |

**Table S5** Future targets of total forest area in China.

| Target year | total forest area (Mha) | arbor forest area (Mha) |
| --- | --- | --- |
| 2014-2018 | 220.45 | 179.89 |
| 2025 | 231.36 (=960 ×24.1%) | 188.79 (=231.36×179.89/220.45) |
| 2030 | 240.00 (=960 ×25.0%） | 195.84 (=240.00 ×179.89/220.45） |
| 2035 | 249.60 (=960 ×26%) | 203.68 (=249.60×179.89/220.45) |
| 2050 | 294.72 (=960 ×30.7%) | 240.50 (=294.72×179.89/220.45) |

**Notes:**

a. According to the “Report on China's Forest Resources (2014-2018)” published by the National Forestry and Grassland Administration in China, the total forest area (including arbor forest, bamboo and shrubbery) is 220.45 Mha, and the high forest area is 179.89 Mha. We suppose the proportion of high forest remain the same in future.

b. Target 2025: The "Fourteenth Five-Year" Plan Outline for the Protection and Development of Forestry and Grassland set a goal of 24.1% forest cover by 2025. Here we use 960 Mha as China’s total land area.

c. Target 2030. the Action Plan for Carbon Dioxide Peaking Before 2030 outlines China’s target to increase forest cover to 25% by 2030.

d. Target 2035: “National Major Project for the Protection and Restoration of Important Ecosystems Master Plan (2021-2035)” sets a goal of 26% of forest cover by 2035.

e. Target 2050: The 2018 National Forestry Department Directors’ Conference sets a goal of 30.7% in 2050 (reach the world average).

**Table S6** Estimates of C stock (PgC), C density and predictions of biomass variation of other studies.

| reference | C sector | Period | C stock (PgC) | C density (Mg/ha) | prediction of future |
| --- | --- | --- | --- | --- | --- |
| (Qin et al., 2024) | AGB | 2020 | 7.62±0.05 | 88.64±0.64 | 15.51± 0.99 Pg C in 2060 and 19.59± 1.36 Pg C in 2100 |
| (Qiu et al., 2020) | AGB | 2020 | 9.23 |  | 13.90 PgC in 2050 |
| (Tang et al., 2018) | AGB | 2010-2015 | 8.38  (No direct estimate, use C density*area 188×10^6^ha) | 89.2±25 | / |
| (Xu et al., 2018) | vegetable carbon storage | 2004-2014 | 11.49 ± 3.18 | / | / |
| (Zhao et al., 2019) | AGB | 2009-2013 | 7.27 | 89.04 | / |
| (Chen et al., 2022) | AGB | 2010 | 9.40 ± 1.45 | / | / |
| (Yao et al., 2018) | AGB | 2010s | 10.75 ± 0.005 | / | 17.44 ± 0.005 Pg C in the 2040s.,1.09 Pg C/decade in the 2040s |

# References

Chen S, Lu N, Fu B, Wang S, Deng L, Wang L. Current and future carbon stocks of natural forests in China. Forest Ecol Manag. 2022;511:120137. doi:10.1016/j.foreco.2022.120137.

Qin J, Liu P, Martin AR, Wang W, Lei Y, Li H. Forest carbon storage and sink estimates under different management scenarios in China from 2020 to 2100. Sci Total Environ. 2024;927:172076. doi:10.1016/j.scitotenv.2024.172076.

Qiu Z, Feng Z, Song Y, Li M, Zhang P. Carbon sequestration potential of forest vegetation in China from 2003 to 2050: Predicting forest vegetation growth based on climate and the environment. J Clean Prod. 2020;252:119715. doi:10.1016/j.jclepro.2019.119715.

Tang X, Zhao X, Bai Y, Tang Z, Wang W, Zhao Y, et al. Carbon pools in China’s terrestrial ecosystems: New estimates based on an intensive field survey. Proc Natl Acad Sci U S A. 2018;115:4021-4026. doi:10.1073/pnas.1700291115.

Xu L, Yu G, He N, Wang Q, Gao Y, Wen D, et al. Carbon storage in China’s terrestrial ecosystems: A synthesis. Sci Rep. 2018;8:2806. doi:10.1038/s41598-018-20764-9.

Yao Y, Piao S, Wang T. Future biomass carbon sequestration capacity of Chinese forests. Sci Bull. 2018;63:1108-1117. doi:10.1016/j.scib.2018.07.015.

Zhao M, Yang J, Zhao N, Liu Y, Wang Y, Wilson JP, et al. Estimation of China’s forest stand biomass carbon sequestration based on the continuous biomass expansion factor model and seven forest inventories from 1977 to 2013. Forest Ecol Manag. 2019;448:528-534. doi:10.1016/j.foreco.2019.06.036.
